# Health service readiness and quality for sick child care: an effective coverage analysis in eight low- and middle-income countries

**DOI:** 10.7189/jogh.15.04085

**Published:** 2025-04-02

**Authors:** Abdoulaye Maïga, Gouda Roland M Mady, Elizabeth A Hazel, Safia S Jiwani, Emily B Wilson, Assanatou Bamogo, Helen W Kiarie, Agbessi Amouzou

**Affiliations:** 1 Department of International Health, Johns Hopkins University Bloomberg School of Public Health, Baltimore, Maryland, USA; 2 Monitoring and Evaluation Division, Ministry of Health, Nairobi, Kenya

## Abstract

**Background:**

Most child deaths can be averted through prompt and appropriate treatment of child illnesses such as pneumonia, diarrhoea, and malaria. However, research has suggested that increases in care seeking do not necessarily mean that quality care is being received. We assessed the service readiness and process quality of curative healthcare during childhood and determined whether children are receiving health services with sufficient quality across countries.

**Methods:**

We linked data from household surveys including the standard Demographic and Health Survey and the Multiple Indicator Cluster Survey to data from facility surveys including the Service Provision Assessment and Health Facility Assessment in Bangladesh, the Democratic Republic of Congo, Haiti, Kenya, Malawi, Nepal, Senegal and Tanzania to estimate the effective coverage of child illness treatment. We assessed the gaps in service availability and coverage, lack of service readiness, missed care opportunities, and inadequate service process, where service readiness and process quality were defined according to global standards with country-specific adaptations. We analysed the service readiness, quality of care, and effective coverage by individual illness and combined illnesses accounting for equity dimensions.

**Results:**

Seven to 42% of children experienced at least one illness. An integrated management of child illnesses (IMCI) service was available in 58–85% of facilities. We found that 55–66% of health facilities in the countries were ready to deliver treatment to sick children. However, the readiness-adjusted contact suggested that child healthcare was mostly sought in facilities with low readiness score, ranging from 15% (Nepal) to 46.0% (Malawi). Health facilities had low diagnostics, supervision, and trained personnel capacity to manage child illnesses. Concerning the quality of care, only 51–60% of the procedures during clinical encounters were in line with standards. Counselling of caretakers had the lowest score, while treatment components had the highest process quality score. Hospitals had higher readiness and process quality scores compared to primary facilities and the private sector. There were, however, large gaps in service readiness and significant inadequate service processes in all countries; 35% (Haiti) to 79% (Bangladesh) of sick children sought care from a health facility, with only 7% (Nepal) to 29% (Malawi) of them actually receiving appropriate treatment. We found large inequalities in care seeking, quality of care, and effective coverage across levels of education and poverty, and places of residence.

**Conclusions:**

A large proportion of facilities did not meet the required capacity to provide IMCI services. The provision of health services has major quality gaps, highlighting the need for strengthening health service access, capacity and quality of care to reach universal child health coverage.

Low- and middle-income countries (LMICs), specifically those in sub-Saharan Africa, have the highest under-five children mortality rates in the world estimated at 71 deaths per 1000 live births [1]. In 2022, countries in sub-Saharan Africa and Southern Asia accounted for more than 80% of the 4.9 million deaths of under-five children in the world [2]. Malaria, diarrhoea, and pneumonia are the major causes of death among children 1–59 months in LMICs, leading to their prioritisation in the integrated management of childhood illnesses (IMCI) strategy [3], which is a comprehensive and integrated approach to reduce preventable mortality among children under five years through preventive and curative care, by improving family and community health practices for health along with strengthening health system capacity to provide quality care [4].

This strategy has been used by many health programmes and interventions aiming to reduce child mortality [5–9]. For this reason, most studies on IMCI include an assessment of child illness treatment coverage. However, poor quality of healthcare has been identified as a possible reason for the persistent high child mortality rates in LMICs, despite recent increases in health intervention coverage [10–12]. Most coverage indicators used for this purpose function as service contact measurements and do not reflect the actual content or quality of services received during clinical encounters. There is evidence that poor-quality care results in more than 8.6 million population deaths per year [13], and that 71% of neonatal deaths, 33% of stillbirths, and 54% of maternal deaths could be averted per year by increasing intervention coverage and improving health service quality [14]. The World Health Organization (WHO) has thus emphasised that quality of care remains a significant challenge, resulting in excess and avoidable mortality, particularly among children [10].

International health organisations and the research community have increasingly called for initiatives to go beyond the contact coverage metric by incorporating quality of care dimensions into measures of coverage, allowing them to understand whether children (and broader populations) are receiving health interventions with sufficient quality to produce a health gain [15–18]. Coverage measures reflecting quality of care, also known as effective coverage, can then be applied across the continuum of care for reproductive, maternal, newborn, child and adolescent health and nutrition services [15,16]. However, only a few studies have analysed effective coverage during childhood.

We aimed to assess the capacity of health facilities to deliver quality treatment and calculate the effective coverage of curative health service during childhood, accounting for the quality of care in eight LMICs. For this purpose, we defined effective coverage as the proportion of sick children in need of treatment who received healthcare with sufficient quality service, likely to result in a positive health outcome. We further wanted to address coverage measurement gaps by establishing a coverage cascade model which highlights gaps in service availability and coverage, lack of service readiness, missed opportunities, and inadequate service process for sick children. Here we focussed on the main illnesses included in the IMCI strategy, which include fever, *i.e.* presumed malaria, diarrhoea, and acute respiratory infections (ARIs), *i.e. *presumed pneumonia.

## METHODS

### Study framework

We used the WHO guidelines and other global IMCI and quality of care frameworks to conceptualise this study. Specifically, the WHO’s guidelines informed the identification of key IMCI interventions and the definition of intervention coverage and quality of care indicators [4,19–21]. Donabedian’s model provided useful information about health service delivery, including the structural quality and process quality [22,23]. We also reviewed the service provision assessment (SPA) and the service availability and readiness assessment (‎SARA)‎ guidelines to identify key IMCI quality tracer items and calculate intervention coverage and quality of care indicators [24–27]. We further performed a literature review to understand the definition of service availability, care seeking, lack of service readiness, missed opportunities, and inadequate service process [15,16,28].

### Study data

We used data from the standard Demographic and Health Survey (DHS) and the Service Provision Assessment (SPA), both conducted by the DHS Program [24,25]. We also included the Multiple Indicator Cluster Survey (MICS) implemented by the United Nations Children's Fund (UNICEF) and the Health Facility Assessment (HFA) surveys implemented by several countries with technical support from the WHO [29–31]. The DHS and MICS data are household-level surveys and contain variables that allow for the assessment of child illnesses and computation of the coverage of child health services and interventions. Meanwhile, SPA and HFA are facility-level surveys that include data for assessing health service availability, readiness, and quality of care. These are sample-based and complete census facility datasets representative at the national level. Here we only included surveys conducted in 2010 or after from countries with a time interval between the most recent household survey and facility survey of no more than three years. This was critical for linking household and facility data sets, and adjusting healthcare coverage rates with accurate quality of care data as facility readiness doesn’t change much within 2–3 years. Eight countries met theses inclusion criteria: Bangladesh, with the 2017–18 DHS and 2017 SPA, the Democratic Republic of Congo (DRC) with the 2017 MICS and 2017–18 SPA, Haiti with the 2016–17 DHS and 2017–18 SPA, Kenya with the 2022 DHS and 2018–19 HFA, Malawi with the 2015–16 DHS and 2013–14 SPA, Nepal with the 2022 DHS and 2021 SPA, Senegal with the 2017–19 DHS and 2019 SPA, and Tanzania with the 2015–16 DHS and 2014–15 SPA [25].

### Analysis of quality of care

We focussed on two dimensions of quality of care: health service readiness, also known as structural quality, and process quality, also known as service provision quality [22,23]. We defined the health service readiness as the availability, in sufficient quantity and quality, of all inputs necessary to provide the service at the time of the visit in the health facility [16]. It thus reflects the capacity of health facilities to provide IMCI service and comprises selected readiness components according to countries standards. These components include the presence of child specific health service, the availability of equipment and supplies, diagnostics, training and supervision of the health staff, and essential medicines and commodities for child curative care in facilities [24,26] (Table S1 in the **Online Supplementary Document**). We calculated the health facility readiness score defined as the proportion of required IMCI readiness tracer items available in the facility. We also computed the readiness-adjusted coverage, defined as the proportion of care seeking from a health facility ready to provide IMCI service. There is no standard operational definition of the process quality; typically, it refers to the provision of health services or care by health personnel according to recommended protocols and standards of the country [15–17]. It includes expected services, protocols, and actions for child illness treatment. The main components of service provision comprised the assessment of the child’s health and health history, physical exams, treatment of the child, and the counselling of the caretakers (Table S1 in the **Online Supplementary Document**).

Health service readiness data were available for all countries, while service provision data have not been collected in Bangladesh and Kenya. Each quality dimension comprises quality components that, in turn, are composed of quality tracer items or indicators used to compute the readiness and process quality scores (Figure S1 in the **Online Supplementary Document**). We calculated the overall score of each of the quality dimensions (service readiness and process quality) using an arithmetic average of the number of tracer items across facilities. The score could range from 0% and 100%, whereby a score of 0% means that the facility does not meet none of the country’s service readiness or process quality standards, while a score of 100% means that the facility meets all required readiness or quality tracer items for child curative healthcare. We also calculated the score for each component based on tracer items within the component. The scores were disaggregated according to the type of facility (hospital *vs*. health centre) and the management authority (public *vs*. private).

### Analysis of effective coverage

We calculated effective coverage rates by linking household and facility data by stratum and adjusting the coverage estimates from household surveys with quality of care scores from facility surveys [17,32]. We formed the strata variable by combining the geographical region and the type of facility and the managing authority. Within each stratum, we multiplied the average care seeking coverage estimate from the household survey by the average quality of care score from facility data to get effective coverage rates.

We constructed an effective coverage cascade based on the framework proposed by Amouzou and colleagues [15] (**Table 1**). We built the coverage cascades for each of the three illnesses and a combined illness indicator. The first step of the cascades refers to the population in need of health service or care. The target population for diarrhoea, fever, and ARI was based on children who experienced episodes of these illnesses in the two weeks preceding the survey interview. In the second step, we assessed whether any care was sought during the illness episode. The third step was service contact, *i.e. *care seeking from a health facility or skilled provider. The fourth step incorporated health service readiness, which is care seeking from a facility ready to manage child illnesses in line with IMCI guidelines. The care seeking coverage (crude coverage) for diarrhoea treatment was defined as for the proportion of children with diarrhoea in the two weeks preceding the survey who received an oral rehydration solution (ORS) intervention. For malaria, we computed the proportion of children with fever taken to formal care and who received a blood testing for malaria. The administration of antibiotics is the recommended treatment of pneumonia. However, it is difficult to accurately assess pneumonia cases through household surveys, and the treatment data are also less reliable [33]. For this reason, we used reported care seeking from a health facility or skilled provider (service contact) as crude coverage indicator for children with ARI/symptoms of pneumonia. Lastly, we calculated the coverage adjusted by process quality as described above. We also applied these steps to the combined three IMCI illnesses indicator.

**Table 1:** Coverage cascades indicators of sick children

	Indicators			
**Cascade level**	**Diarrhoea**	**Fever/malaria**	**ARI/pneumonia**	**IMCI**
Population in need	Child had diarrhoea	Child had fever/symptoms of malaria	Child had ARI/symptoms of pneumonia	Child had diarrhoea or fever/malaria or ARI/symptoms of pneumonia
Care seeking (any)	Care seeking from any sources			
Service contact	Care-seeking from a health facility or skilled provider			
Readiness-adjusted contact	Care seeking from a facility ready to manage illness in line with IMCI guidelines			
Crude coverage of intervention	ORS	Blood testing for malaria	Service contact	ORS, malaria testing, service contact for ARI
Quality-adjusted coverage	Intervention delivered according to standards			

ARI – acute respiratory infection, ORS – oral rehydration solution

We performed an equity analysis, calculating and disaggregating the estimates at country level, by place of residence, wealth quintile, education level and age of the mother/caretaker, sex and age of the child. We carried out this step according to the household and health facility surveys’ complex designs, accounting for survey weights, clustering, and stratification. We used the household survey data as the master data set to which health facility survey data were linked. We used the household sampling design to calculate the uncertainties including standard errors and confidence intervals for child specific coverage indicators. Uncertainties about facility specific indicators (*e.g.* service availability, readiness and process quality score) were based on health facility assessment sampling design. We used uncertainties and the statistical precision of estimates to assess whether the differences were statistically significant. We used Stata, version 16.1 (StataCorp LLC., College Station, Texas, USA) for the analyses.

## RESULTS

### Child illnesses and care seeking

The proportion of children under five year of age who experienced any of the three IMCI diseases in the past two weeks prior to the survey varied from 7.4% (95% confidence interval (CI) = 6.8–8.1) in Bangladesh to 41.6% (95% CI = 40.4–42.9) in Malawi, with a median of 27.4% (Table S2 in the **Online Supplementary Document**). Fever was the most prevalent illness (median prevalence: 18.7%) followed by diarrhoea (median prevalence: 14.2%) and ARIs (median prevalence: 3.7%).

The proportion of ill children receiving formal care similarly differed across countries, going from 82.9% (95% CI = 79.3–86.0) in Bangladesh to 29.0% (95% CI = 27.5–30.5) in Senegal, and malaria testing was particularly low across countries (**Table 2**). The lowest proportion of children with diarrhoea who received ORS was in Senegal. Bangladesh had highest care seeking rates for most interventions, with more than 80% of children receiving ORS for diarrhoea or having sought care for ARIs. In contrast, care seeking rates for ARIs was below 50% in the DRC (34.7%; 95% CI = 26.5–44.0), Haiti (35.4%; 95% CI = 30.4–40.8) and Senegal (49.4%; 95% CI = 45.1–53.6).

**Table 2:** Formal care seeking/treatment for sick children

Country	Care seeking/treatment	% (95% CI)	Country	Care seeking/treatment	% (95% CI)
Bangladesh	Diarrhoea – ORS	83.3 (78.9–87)	Malawi	Diarrhoea – ORS	64.7 (62.6–66.9)
Bangladesh	Fever – malaria test	NA	Malawi	Fever – malaria test	52 (49.7–54.3)
Bangladesh	ARI – care seeking	81.1 (75.6–85.7)	Malawi	ARI – care seeking	69.3 (64.8–73.4)
Bangladesh	IMCI – care seeking	82.9 (79.3–86.0)	Malawi	IMCI – care seeking	63 (61.2–64.7)
DR Congo	Diarrhoea – ORS	24.1 (20.8–27.6)	Nepal	Diarrhoea – ORS	38.4 (32.9–44.2)
DR Congo	Fever – malaria test	22.3 (19.7–25.1)	Nepal	Fever – malaria test	NA
DR Congo	ARI – care seeking	34.7 (26.5–44.0)	Nepal	ARI – care seeking	74.6 (61.6–84.3)
DR Congo	IMCI – care seeking	26.3 (23.9–28.8)	Nepal	IMCI – care seeking	43.1 (37.9–48.5)
Haiti	Diarrhoea – ORS	39.3 (35.7–43.0)	Senegal	Diarrhoea – ORS	28.2 (26.5–29.9)
Haiti	Fever – malaria test	NA	Senegal	Fever – malaria test	15.4 (14.0–16.9)
Haiti	ARI – care seeking	35.4 (30.4–40.8)	Senegal	ARI – care seeking	49.4 (45.1–53.6)
Haiti	IMCI – care seeking	39.9 (36.7–43.2)	Senegal	IMCI – care seeking	29 (27.5–30.5)
Kenya	Diarrhoea – ORS	48.3 (45.4–51.2)	Tanzania	Diarrhoea – ORS	44.8 (41.0–48.6)
Kenya	Fever – malaria test	33.4 (31.3–35.6)	Tanzania	Fever – malaria test	35.9 (32.6–39.4)
Kenya	ARI – care seeking	82.3 (77.3–86.5)	Tanzania	ARI – care seeking	55.5 (49.0–61.7)
Kenya	IMCI – care seeking	46.1 (44.1–48.1)	Tanzania	IMCI – care seeking	44.5 (41.7–47.2)

ARI – acute respiratory infection, CI – confidence interval, IMCI – integrated management of childhood illness, NA – not applicable, ORS – oral rehydration solution,

Public health centres were the principal source of care in most countries (the DRC, Kenya, Malawi, Senegal, and Malawi). They represented 12% (Nepal and Haiti) to 40% (Senegal and Malawi) of the source of care for child illnesses. We found different patterns in Bangladesh and Nepal (South-East Asia countries) where the private sector played a greater role in curative health services for children, with private health centres and pharmacies/depots being the main sources. There was a non-negligible proportion of sources of care which were undetermined, particularly in Malawi. Conversely, there was a marginal proportion of care seeking from community health workers, ranging from 0.7% (95% CI = 0.4–1.0) in Senegal to 4.2% (95% CI = 2.9–5.9) in Tanzania (Table S3 in the **Online Supplementary Document**).

### Health facility IMCI service readiness

The overall health facility readiness score was moderate and similar across countries, ranging from 55.0% (95% CI = 54.2–55.8) in Bangladesh to 65.7% (95% CI = 65.1–66.3) in Kenya. Hospitals, particularly from the public sector, were more ready to provide IMCI services in all countries. Public hospitals had more than 70% of readiness score in Bangladesh, Kenya, Malawi, Senegal, and Tanzania. Private health centres had lower readiness score compared to public health centres across countries, particularly in Senegal (20 percentage points gap) and Nepal (34 percentage points gap).

The scores varied according to the readiness components. Most facilities had IMCI services. About 80% of facilities had IMCI services in all countries, except in Bangladesh (67.4%; 95% CI = 66.3–68.4) and Haiti (73.9%; 95% CI = 72.5–75.3). The scores for the remaining components were similar in Bangladesh. The DRC had most of the diagnostics items particularly in public hospitals (92.2%; 95% CI = 90.2–94.3) and private hospitals (94.7%; 95% CI = 93.0–96.4). Kenya, meanwhile, had the lowest score on availability of IMCI diagnostics (23.4%, 95% CI = 22.5–24.4). There were large gaps in the readiness score by component across the categories of health facilities. For example, 90% of diagnostics items on average were available in public and private hospitals against 37.6% (95% CI = 32.4–42.8) and 57.7% (95% CI = 54.0–61.5) in public and private health centres in Haiti, respectively. We observed similar patterns in Malawi, Senegal, and Tanzania. The supply items scores were lower in Kenya (55.6%; 95% CI = 54.9–56.3), Haiti (55.1%; 95% CI = 53.9–56.4), and Bangladesh (54.5%; 95% CI = 53.4–55.5). Fewer health workers have been trained in management of IMCI in the last two years and have received a supervision in the past six months before the survey in Haiti, Malawi, Nepal, Senegal, and Tanzania (Table S4 in the **Online Supplementary Document**).

### Quality of IMCI services

More than half of process quality tracer items were observed during IMCI clinical encounters in all countries. The process quality refers to procedures, protocols, information, and actions provided by the health provider during health assessment, physical exam, treatment and counselling of the caretaker. Tanzania (60.1%; 95% CI = 59.6–60.6) and Senegal (51.6%; 95% CI = 50.7–52.6) had the highest and lowest process quality scores, respectively. Hospitals (mean score: 68.1%) contributed most to the high score in Tanzania while the low score in private facilities (mean score: 46.4%) pulled Senegal’s overall score down.

The counselling of child caretakers had the lowest process quality component score, at 18.6% (95% CI = 16.7–20.4) for all facilities and 9.3% (95% CI = 5.0–13.5) in private health facilities in Senegal. On the other hand, Tanzania’s health workers met 43.2% (95% CI = 42.1–44.2) and 54.1% (95% CI = 51.6–56.6) of counselling items in total and in public hospitals respectively. The differences between the remaining process quality components (health assessment, physical exam, treatment) were small, except in Senegal, where the treatment score was particularly low (37.1%; 95% CI = 34.6–39.7). By contrast, the treatment component had the highest score in the other countries, nearly at or above 60%. The overall process quality score showed higher scores among hospitals in comparison to health centres. However, the analysis by quality component revealed higher scores for treatment in private health centres in the DRC and Haiti in particular (Table S5 in the **Online Supplementary Document**).

### Effective coverage of child illness management

**Figure 1:**
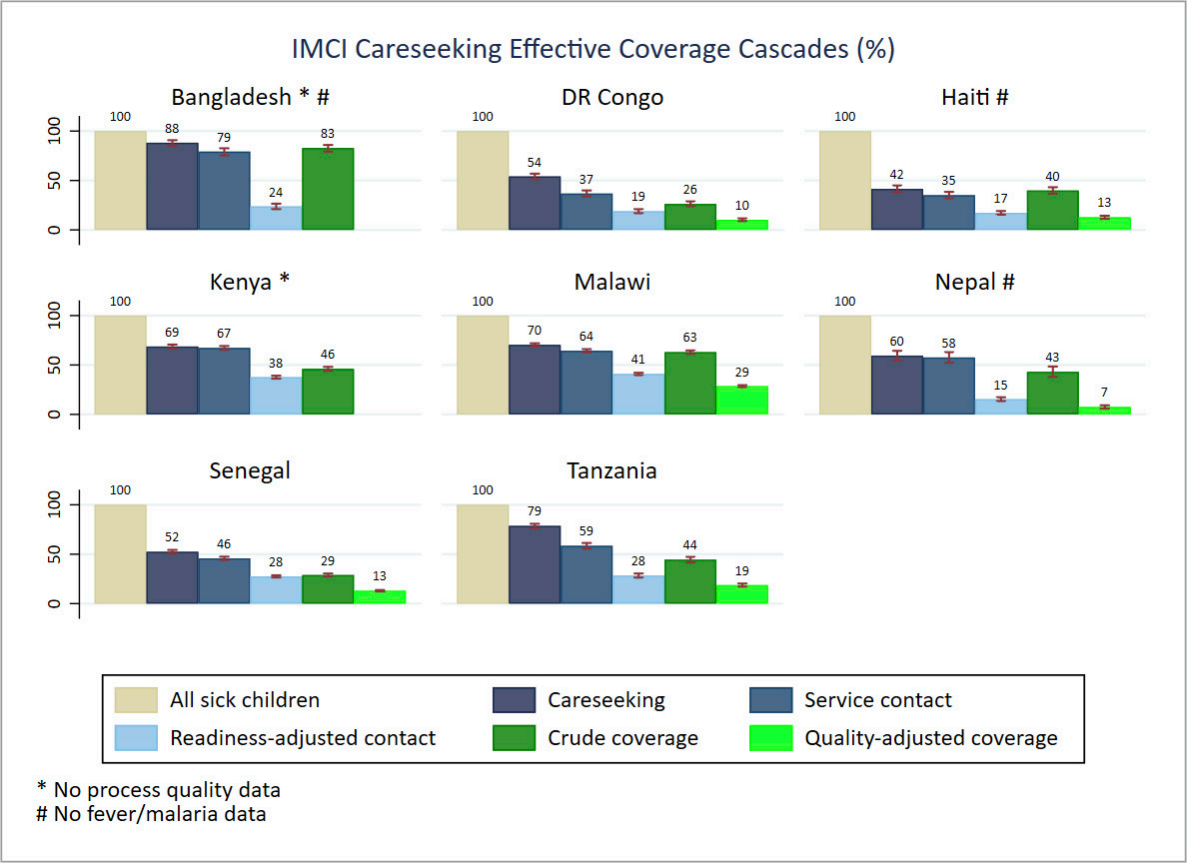
IMCI care seeking effective coverage cascades.

A high proportion of sick children were taken to any care provider (care seeking) in Bangladesh (88.2%, 95% CI = 85.0–90.8) and Tanzania (78.7%; 95% CI = 76.4–80.8). In contrast, only 41.5% (95% CI = 38.1–45.0) and 52.5% (95% CI = 50.6–54.4) of sick children sought care in Haiti and Senegal, respectively. The sources of care seeking were mainly health facilities and/or skilled providers (service contact). However, the difference between the care seeking and service contact rates showed significant missed opportunities in Bangladesh, the DRC, Malawi, Senegal, and Tanzania, where 8%, 17%, 6%, 6%, and 20% of care seeking, respectively, were not from a health facility or skilled provider. In general, the health facilities visited had low service readiness scores, where the readiness-adjusted contact highlighted that only 15.4% (95% CI = 13.4–17.3) and 17.3% (95% CI = 15.4–19.1) of sources of care visited were ready to provide IMCI service in Nepal and Haiti respectively. The highest proportions of readiness-adjusted contact were in Malawi (40.9%, 95% CI = 39.7–42.1) and Kenya (37.7%, 95% CI = 36.2–39.2). The proportion ranged between 19% and 29% for the remaining countries. The comparison of the crude coverage and quality-adjusted coverage highlighted significant inadequate service processes across countries with available data. The data suggested that 34% and 35% of IMCI service did not meet process quality standards in Malawi and Nepal (**Figure 1**). Similar inadequate service process was observed in Tanzania (25%), Haiti (27%), the DRC (16%), and Senegal (16%).

**Figure 2:**
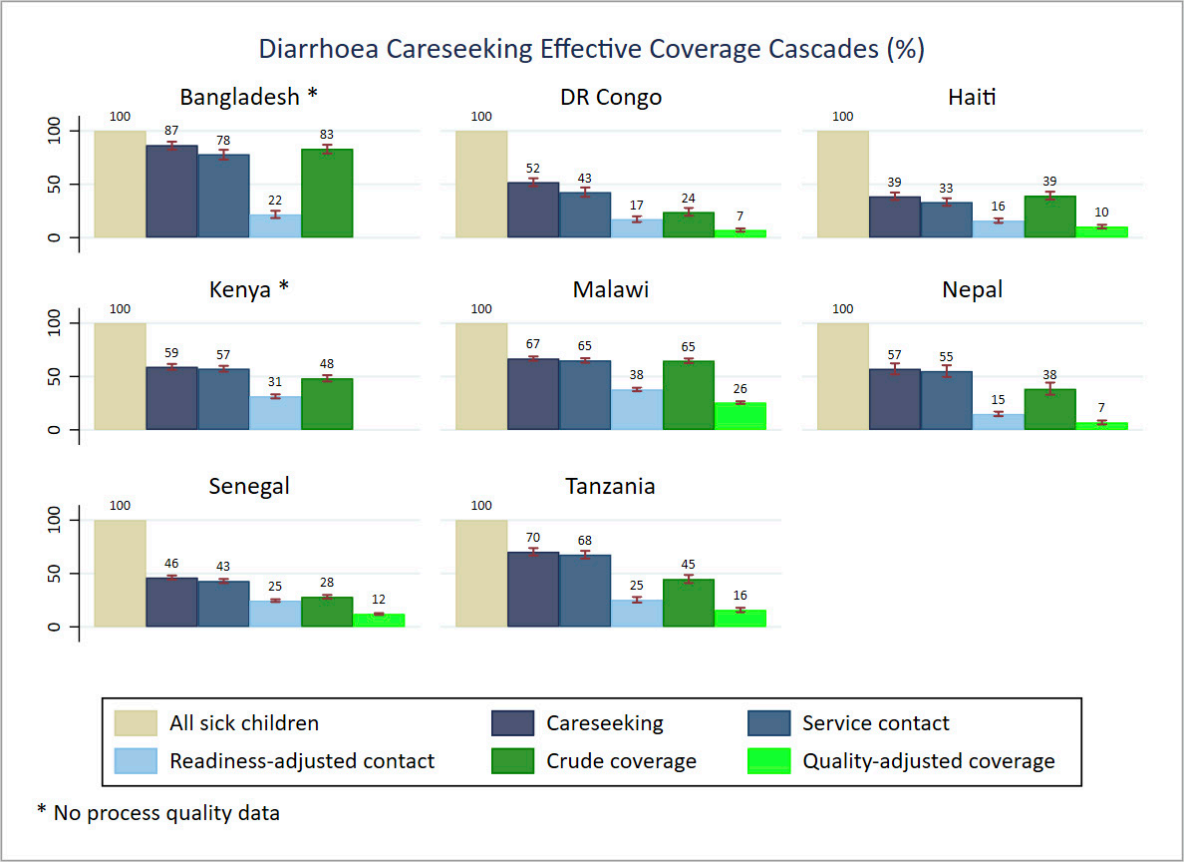
Diarrhoea case management effective coverage cascades.

**Figure 3:**
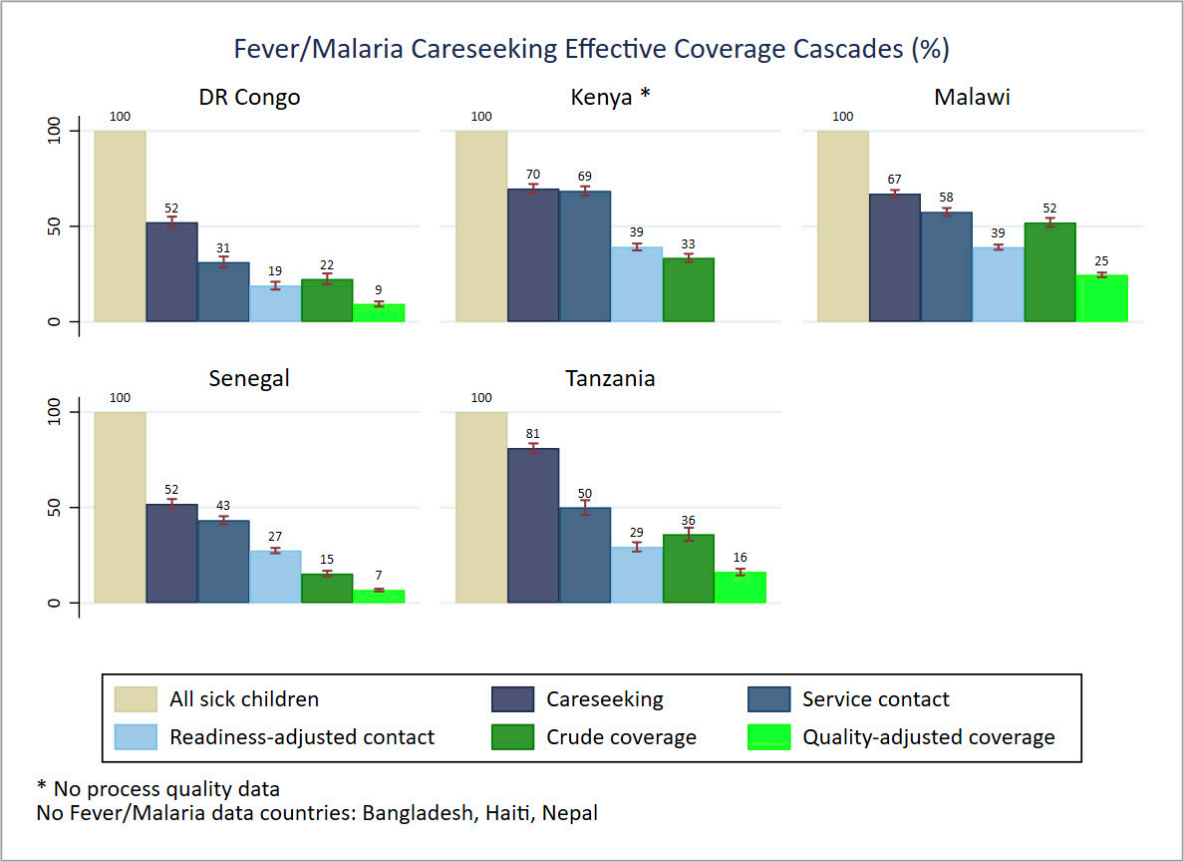
Fever/malaria case management effective coverage cascades.

**Figure 4:**
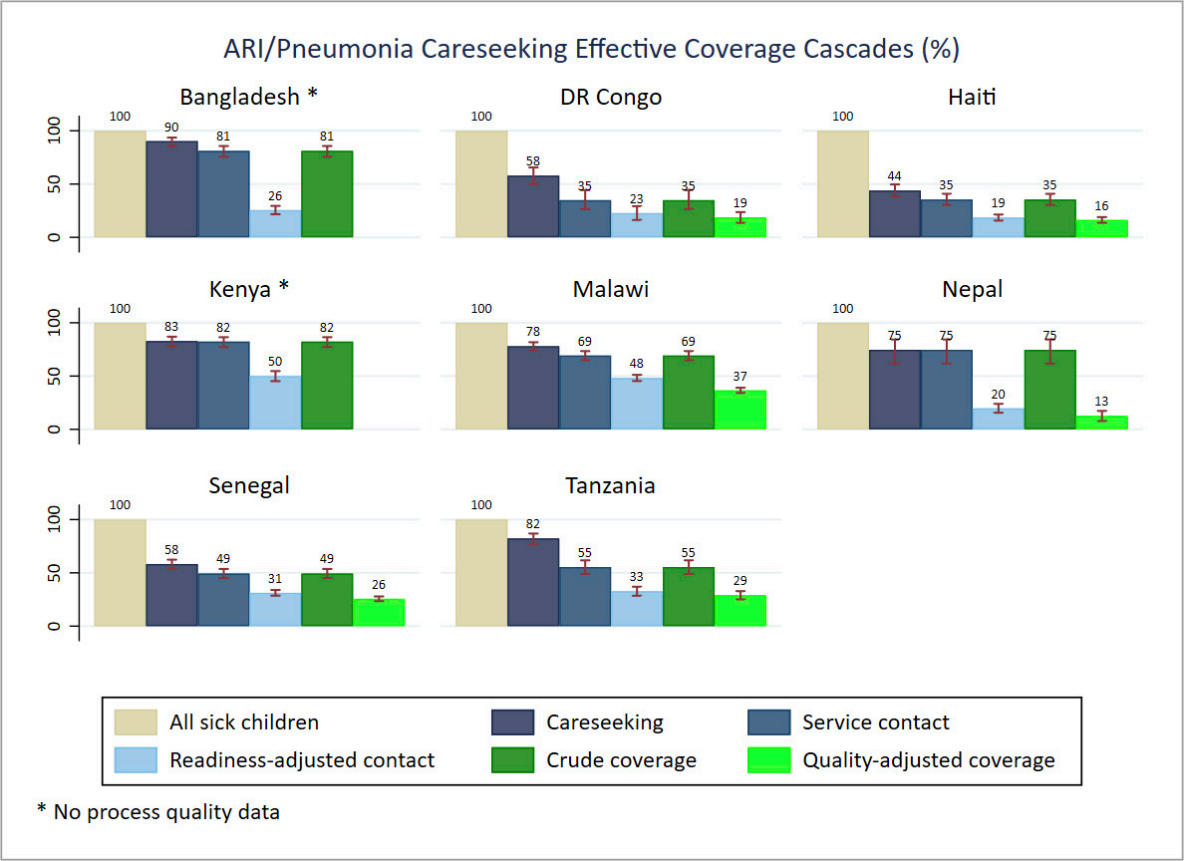
ARI/symptoms of pneumonia case management effective coverage cascades.

We found different patterns of effective coverage by individual illness. In general, care seeking from any sources (care seeking) and from a health facility (service contact) were higher for ARIs compared to fever and diarrhoea, which ranked second and third, respectively. The readiness-adjusted contact data suggested higher proportions of care seeking from health facilities ready to manage symptoms of pneumonia, particularly in Kenya (50.0%; 95% CI = 45.2–54.7) and Malawi (48.2%; 95% CI = 45.3–51.1). The facility readiness score for the remaining countries ranged from 18.5% (95% CI = 15.7–21.3) in Haiti to 32.7% (95% CI = 28.5–36.9) in Tanzania for care seeking related to symptoms of pneumonia. A lower proportion of facilities were ready for management of diarrhoea cases. Bangladesh (83.3%, 95% CI = 78.9–87.0), Kenya (48.3%, 95% CI = 45.4–51.2) and Malawi (64.7%, 95% CI = 62.6–66.9) had higher crude coverage rates of diarrhoea management compared to Haiti (39.3%, 95% CI = 35.7–43.0) and Senegal (28.2%, 95% CI = 26.5–29.9). The proportion of children receiving health treatment (crude coverage) by illness was generally low across countries. Comparing the crude coverage to the proportion who received the intervention according to standards (quality-adjusted coverage), we found variable and inadequate service processes across all illnesses and countries. The ratio of both proportions revealed that the proportion of children with diarrhoea receiving ORS was 2.3 times (Senegal) lower than the proportion who had a service contact. In Nepal, the process quality gap for diarrhoea management was much higher (5.6 times). Regarding the management of symptoms of pneumonia, the proportion who received the service according to standards was about two times lower than those who received the service across countries, except for Nepal (5.9 times). The quality gap ratio showed a similar pattern for management of symptoms of malaria, about half of the children with fever had their blood taken for malaria testing (**Figure 2****,** **Figure 3****,** **Figure 4**).

### Equity stratification of effective coverage of child illness management

In general, there were no significant differences of care seeking by place of residence, except in Senegal and Haiti, where the care seeking from any sources was 13 and 16 percentage points lower in rural areas in comparison of urban areas, respectively. Care seeking from a health facility or skilled provider (service contact) was also lower in rural areas in both countries (−9 and −15 percentage points for Senegal and Haiti, respectively), while the service contact gap was much higher in Tanzania (−17 percentage points). Only these three countries were also characterised by a rural disadvantage regarding the proportions of sources of care ready to provide IMCI services (readiness). The proportion of sick children who received a health intervention (crude coverage) was 13 lower in rural areas in Haiti and 20 percentage points lower in those in Tanzania. We also found poorer process quality in rural areas in both countries. There were no differences by place of residence across the cascade components in Bangladesh, the DRC (except crude coverage), Kenya, Malawi (except care seeking), and Nepal (Table S6 in the **Online Supplementary Document**).

The analysis by wealth quintile showed lower proportions of care seeking among children from poorest households in the DRC (−10 percentage points), Haiti (−12 percentage points), and Senegal (−16 percentage points) compared to the richest group. Richest mothers and caretakers were more likely to seek care from a health facility or a skilled provider in Haiti (10 percentage points), Senegal (12 percentage points), and Tanzania (14 percentage points) compared to poorest groups. Regarding service readiness, a higher proportion of children from richest households had visited a health facility ready for management of child illnesses in Bangladesh (10 percentage points), Senegal (7 percentage points), and Tanzania (10 percentage points). There were no differences of crude coverage by wealth quintiles, except in Tanzania, with crude coverage being 16 percentage points coverage higher among children from the richest group compared to those in poorest households. Furthermore, the quality-adjusted coverage showed poorer service provision among children from poorest households in the DRC (−5 percentage points), Haiti (−5 percentage points), and Tanzania (−7 percentage points) compared to children from the richest quintile. We found no differences by wealth quintile for all estimates in Kenya, Malawi and Nepal (Table S7 in the **Online Supplementary Document**).

The analysis showed no statistical difference by level of education for all cascades items in Bangladesh and Nepal. The proportion of children who sought any care along with the proportion seeking care from a health facility were 10 percentage points higher among women who had a secondary education level compared to those with no education in Kenya. These proportions were 7 percentage points higher among women who completed the primary level compared to those with no education in Malawi. We found a difference of crude coverage by education level in the DRC only, at 30.3% (95% CI = 27.2–33.6) among women of secondary level and 23.2% (95% CI = 20.0–26.7) for those of primary education level. The proportion of care seeking from a facility that was ready was 6 percentage points higher among mothers/caretakers of secondary or plus level compared to those of primary level and 8 percentage points higher than the non-educated group in Haiti. Similar differences were observed in Senegal and Tanzania. We found positive relationships between the education level and coverage estimates in Haiti, Senegal and Tanzania, with intervention coverage rates increasing according to the level of education. These countries were also characterised by poorer quality of care among less educated women. (Table S8 in the **Online Supplementary Document**).

There were no differences by age of mother/caretaker and by sex of the child across all coverage cascade dimensions across all the countries (Tables S9–11 in the **Online Supplementary Document**). There were also no significant differences by age of the child, except for marginal ones in Nepal (for crude coverage), Senegal (for care seeking and service contact), and Tanzania (for service contact and crude coverage).

## DISCUSSION

We found that LMICs continue to experience high child mortality burden, despite substantial increases in child health service coverage. Low-quality health systems are highlighted as a critical challenge to achieve universal health coverage and reduce mortality [10,11]. However, few studies have accounted for the quality of care in assessing child health coverage. The lack of data on quality of care partly explains this situation. Here we sought to assess the quality of care, the coverage of curative child health services, and whether sick children are receiving quality services according to standards across several settings. While doing so, we focussed on diarrhoea, malaria, and pneumonia due to the lack of data (irrespective of the quality thereof) and the fact that they are among the top causes of child death in LMICs [3]. These three illnesses are also those mainly assessed in IMCI services. We assessed the capacity of health facilities to provide IMCI services and to which extent the services reflect required standards.

On average, facility readiness scores indicate insufficient readiness of facilities to deliver IMCI service, with only 55–66% of facilities being ready across the eight countries. There were also large readiness gaps between hospitals and health centres, favouring the former. The public-private readiness gaps we observed here probably stemmed from the lack of trained health personnel and adequate supervision in private facilities, which also usually had an insufficient supply of diagnostics and essential medication for sick children. This finding calls for appropriate actions by increasing the number of qualified health personnel, improving training and supervision of the personnel, and improving the supply chain system to ensure the availability of medicines and improve the overall health system readiness [34]. There were no large differences between the process quality components, except the counselling of child caretakers during clinical encounters, which received particularly low scores. Child treatment, meanwhile, had higher process component scores. Similarly to the facility readiness score, the overall process quality showed higher scores among hospitals than health centres. However, the treatment score was high in private health centres in some of the countries, such as the DRC and Haiti.

The effective coverage cascade highlighted large gaps of service readiness and significant inadequate service provision in all of the eight countries. We found that a large proportion of health facilities visited for care seeking did not meet the required capacity to provide IMCI services, while a large proportion of the provided services did not meet quality standards of healthcare for sick children. That was true for both combined and individual illnesses. Our findings are consistent with evidence from other studies and highlight the need for ensuring complete components of IMCI services and quality service provision according to standards [17,35,36].

The level of education of the mother/caretaker, the level of poverty, and the place of residence were the most discriminant factors for care seeking, service coverage, and quality of care. Rural and poorer populations generally have lower education levels, lower coverage of health infrastructures and personnel, difficult living conditions, and insufficient knowledge of good health practices or behaviours [37,38]. This is consistent with other evidence which showed systematic pro-educated, pro-rich, and pro-urban inequalities and highlighted the importance of these dimensions for supply and demand generation of care during childhood [35,39,40]. However, we should note that the differences by age of the mother and child’s characteristics (age and sex) were marginal. We otherwise found significant differences for most of the equity dimensions in Haiti, Senegal, and Tanzania. The DRC constituted the second equity group with significant differences by place of residence and wealth quintile. There were no or little inequalities in the remaining countries (*i.e. *Bangladesh, Kenya, Malawi, and Nepal) which represented the third equity group. However, the service coverage and quality of care were particularly poor in some of the countries with low inequalities, such as Nepal.

The limitations of our study mostly come from data availability. Specifically, we used children with fever as proxy for malaria and blood taken from finger/heel for testing of malaria as proxy of process quality due to lack of data on actual malaria cases. This approach was, however, justified by the fact that malaria testing is part of the IMCI protocol in malaria-endemic countries. We otherwise may expect a positive bias regarding the number of children with pneumonia, as we used symptoms of pneumonia as a pneumonia proxy, and care seeking from a health facility or skilled provider as proxy for pneumonia treatment. A validation study on care seeking and treatment of pneumonia highlighted the difficulty of accurately assessing pneumonia cases through household surveys. In turn, antibiotics data for treatment of pneumonia, which is the appropriate protocol, were found to be less reliable [33]. Process quality data were not available in Bangladesh and Kenya, and malaria-specific analysis was not applicable in Bangladesh, Haiti, and Nepal, which are classified as non-malaria-endemic countries [41].

Moreover, the health facility surveys whose data we used here did not include questions about offsite testing, while primary health centres which do not have onsite laboratories and diagnostic capacity may send specimens elsewhere for testing. This could partly explain the diagnostic gap between hospitals and health centres. In contrast, primary health centres have typically lower service readiness compared to hospitals, which can constitute real barriers to access comprehensive and quality care [17]. There was a high proportion of care seeking from non-facility providers in some countries, namely pharmacies and depots, preventing us from linking care seeking data from household surveys to health facility service readiness and process quality data, since data from non-facility providers were not available in facility surveys. Given that pharmacies and depots are not primary sources of child curative healthcare in these settings, assigning a null score for service readiness and process quality would not have caused any major bias. Regarding community health workers, we expect the bias due to the lack of readiness and process quality data to be marginal, since the proportion of care seeking from those sources was very low, ranging from 0.7% (95% CI = 0.4–1.0) in Senegal to 4.2% (95% CI = 2.9–5.9) in Tanzania. However, the effective coverage rates may have been underestimated in Malawi, where a non-negligible proportion of sources of care were undetermined. This is a multi-country study covering West and Eastern Africa, South-East Asia, and Caribbean countries. However, these countries are not representative of all LMICs and regions to which they belong, making the generalisation of the findings beyond the specific contexts studied difficult.

However, these limitations do not negate our findings or the strengths of our research. This is one of the first studies to assess the effective coverage of child illness treatment services in a systematic way and across multiple countries. It thus contributes to improving coverage measurement by accounting for health facility service readiness and quality of care. We have also developed a coverage cascade model that can help with assessing gaps in health service readiness, coverage, and quality. We precisely designed this study to reflect health facility service readiness and quality of care, as outlined in the WHO’s guidelines on management of child illness, the WHO’s and United States Agency for International Development’s definition of health service readiness, and generally accepted standards and protocols for IMCI services [4,19–21]. Both household and facility surveys we used here come from standard survey programmes (DHS and MICs) that are well-standardised for comparison across counties. We included household and facility surveys carried out on average one year apart to get similar reference periods for both sources, *i.e.* DHS and MICS (for service demand) and SPA and HFA (for service supply). Furthermore, data were collected for illnesses and care seeking during the two weeks prior to the day of the household interview, minimising recall bias and data quality issues. We analysed the coverage of sick children service while accounting for both health facility service readiness and quality of care.

Furthermore, we addressed the service readiness, process quality, and coverage for IMCI in general and for individual diseases. Inequalities affecting healthcare access, however, are complex and multilevel (*e.g.* contextual, household, and individual levels), comprising geographic, cultural, social, demographic, institutional, and economic factors. The equity dimensions we used here reflect the complexity of inequalities based on data availability. However, further studies targeting sociocultural, institutional, and other socioeconomic barriers to healthcare access would help use better understand any underlying factors on the demand and supply sides and subsequently tailor health interventions and programmes targeting the most disadvantaged populations.

## CONCLUSIONS

The capacity of health facilities to deliver healthcare to sick children according to accepted standards remains a major issue in most health facilities in LMICs. This leads to low performance of health facilities, significant missed opportunities, and large gaps in intervention coverage and quality of care gaps. Our findings highlight suboptimal readiness of health facilities and poor process quality undermining accelerated progress in reducing child mortality in LMICs. Rapid progress toward the Sustainable Development Goals for health in the future must address severe quality of care gaps in the treatment of most killer diseases among children by revamping the IMCI implementation strategy. Our study also reinforces calls for more equitable and socially compatible health policies in terms of service supply and demand generation among less educated, poorer populations and those living in rural areas. This, in turn, requires strengthening the training and supervision of health personnel to address IMCI issues. There is also a need to strengthen facility diagnostic capacity, mainly for primary health centres. The private sector, which contributes significantly to service delivery, also requires particular attention given the poor service readiness and quality of care in a few countries. In view of this, strengthening the healthcare system through the health workforce, and service capacity, quality and access appear as a prerequisite and priority to achieving the goal of universal child health coverage in LMICs.

## Additional material


Online Supplementary Document
