# Supplementary material for: Health service readiness and quality for sick child care: an effective coverage analysis in eight low- and middle-income countries
Source: J Glob Health. 2025 Apr 2;15:04085. doi: 10.7189/jogh.15.04085 (PMC11961055; doi:10.7189/jogh.15.04085)
Supplement: Online Supplementary Document [file jogh-15-04085-s001.pdf]

Supplement to: Maïga A, Mady GRM, Hazel EA, Jiwani SS, Wilson EB, Bamogo A, Kiarie HW, Amouzou A. Health service readiness and quality for sick child care: an effective coverage analysis in eight low- and middle-income countries. J Glob Health. 2025;15:04085.

Table S1. Readiness and process quality data structure

QUALITY OF CARE DOMAIN	QUALITY OF CARE SUB-DOMAIN	QUALITY OF CARE TRACER ITEMS
**SERVICE READINESS**		
	**AVAILABILITY OF HEALTH SERVICE FOR SICK CHILDREN**	
Service readiness	Availability of child health service	Availability of 24-hour service
Service readiness	Availability of child health service	Availability of Preventive and curative care for children under 5
Service readiness	Availability of child health service	Availability of Diagnosis and treatment of malaria
Service readiness	Availability of child health service	Availability of Treatment of acute respiratory infections in children
Service readiness	Availability of child health service	Availability of Malnutrition diagnosis and treatment
Service readiness	Availability of child health service	Availability of Vitamin A supplementation
Service readiness	Availability of child health service	Availability of Iron supplementation
Service readiness	Availability of child health service	Availability of Zinc supplementation
Service readiness	Availability of child health service	Availability of Growth monitoring
Service readiness	Availability of child health service	Availability of Treatment of pneumonia
Service readiness	Availability of child health service	Availability of Administration of amoxicillin for the treatment of pneumonia in children
	**AVAILABILITY OF EQUIPMENT & SUPPLIES FOR SICK CHILDREN**	
Service readiness	Availability of equipment and supplies	Emergency transport
Service readiness	Availability of equipment and supplies	Child scale observed, functioning
Service readiness	Availability of equipment and supplies	Infant scale observed, functioning
Service readiness	Availability of equipment and supplies	Length/height measuring equipment observed, functioning
Service readiness	Availability of equipment and supplies	Thermometer observed, functioning
Service readiness	Availability of equipment and supplies	Stethoscope observed, functioning
Service readiness	Availability of equipment and supplies	Growth chart observed
Service readiness	Availability of equipment and supplies	Timer or watch with seconds hand observed, functioning
Service readiness	Availability of equipment and supplies	Calibrated measuring jar for ORS observed
Service readiness	Availability of equipment and supplies	Cup & spoon observed
	**AVAILABILITY OF DIAGNOSTICS FOR SICK CHILDREN**	
Service readiness	Availability of diagnostics	Malaria test
Service readiness	Availability of diagnostics	Stool microscopy
	**AVAILABILITY OF MEDICINES & COMMODITIES FOR SICK CHILDREN**	
Service readiness	Availability of medicines & commodities	Artemisinin-based combination therapies (ACTs)
Service readiness	Availability of medicines & commodities	Amoxicillin syrup/suspension observed, at least 1 valid
Service readiness	Availability of medicines & commodities	Co-trimoxazole suspension observed, at least 1 valid

QUALITY OF CARE DOMAIN	QUALITY OF CARE SUB-DOMAIN	QUALITY OF CARE TRACER ITEMS
Service readiness	Availability of medicines & commodities	Antibiotic Gentamicin injectable
Service readiness	Availability of medicines & commodities	Antibiotic ampicillin
Service readiness	Availability of medicines & commodities	Penicillin injection
Service readiness	Availability of medicines & commodities	Quinine injection
Service readiness	Availability of medicines & commodities	ORS
Service readiness	Availability of medicines & commodities	Zinc
	**TRAINING & SUPERVISION OF HEALTH PERSONNEL IN CHILD ILLNESS MANAGEMENT**	
Service readiness	Training & supervision of health personnel	Guidelines for IMCI observed
Service readiness	Training & supervision of health personnel	At least one health worker trained in management of IMCI in the last two years
Service readiness	Training & supervision of health personnel	At least one health worker trained in management of malaria in past 24 months
Service readiness	Training & supervision of health personnel	At least one health worker trained in management of diarrhoea in past 24 months
Service readiness	Training & supervision of health personnel	At least one health worker trained in management of acute respiratory infections in the last two years
Service readiness	Training & supervision of health personnel	At least one health worker trained in micronutrient deficiencies and/or nutritional assessment in the last two years
Service readiness	Training & supervision of health personnel	At least one health worker trained in paediatric HIV/AIDS in the last two years
Service readiness	Training & supervision of health personnel	At least one health worker received supervision in past six months
Service readiness	Training & supervision of health personnel	At least one health worker received supervision with observation in past six months
**PROCESS QUALITY**		
	**ASSESSMENT OF SICK CHILD HEALTH HISTORY DURING CLINICAL ENCOUNTER**	
Process quality	Assessment of health history	Assessment of fever symptoms
Process quality	Assessment of health history	Assessment of cough or difficult breathing symptoms
Process quality	Assessment of health history	Assessment of diarrhoea symptoms
Process quality	Assessment of health history	Assessment of ear pain
Process quality	Assessment of health history	Assessment whether the child is unable to drink or breastfeed (danger signs)
Process quality	Assessment of health history	Assessment whether child vomits everything (danger signs)
Process quality	Assessment of health history	Assessment whether child had convulsions with this illness (danger signs)
Process quality	Assessment of health history	Assessment of normal breastfeeding or normal feeding habits/practices when the child is not ill
Process quality	Assessment of health history	Assessment of breastfeeding or feeding habits/practices for the child during the illness episode
	**PHYSICAL EXAM OF SICK CHILD HEALTH DURING CLINICAL ENCOUNTER**	

QUALITY OF CARE DOMAIN	QUALITY OF CARE SUB-DOMAIN	QUALITY OF CARE TRACER ITEMS
Process quality	Physical exams	Checks for fever with thermometer or by touching the child
Process quality	Physical exams	Checks for palmar or conjunctival pallor
Process quality	Physical exams	Child weighted
Process quality	Physical exams	Child weight plotted on growth chart
Process quality	Physical exams	Child's health card was checked (only for children with a health card)
Process quality	Physical exams	Count of child respiration (breaths) for 60 seconds
Process quality	Physical exams	Checks for child's skin turgor for dehydration
	**COUNSELING OF SICK CHILD CARETAKER DURING CLINICAL ENCOUNTER**	
Process quality	Counselling of caretaker	Counselling on giving extra fluids to child during the illness
Process quality	Counselling of caretaker	Counselling on continued feeding of the child during the illness
Process quality	Counselling of caretaker	Counselling on danger signs/symptoms for immediate consultation
	**TREATMENT PROVIDED/PRESCRIBED TO CHILD DURING CLINICAL ENCOUNTER**	
Process quality	Treatment of sick children	Child with malaria diagnosis receiving an antimalarial
Process quality	Treatment of sick children	Child with dehydration diagnosis receiving oral rehydration treatment (ORT)
Process quality	Treatment of sick children	Child with acute or persistent diarrhoea diagnosis prescribed/received Zinc
Process quality	Treatment of sick children	Child with pneumonia diagnosis receiving Amoxicillin or other antibiotics
Process quality	Treatment of sick children	Child with pneumonia diagnosis receiving amoxicillin
Process quality	Treatment of sick children	Child with pneumonia diagnosis receiving Augmentin
Process quality	Treatment of sick children	Child with severe classification/diagnosis referred

Table S2. Prevalence of child illness

Country	Illness	%	95% CI		Country	Illness	%	95% CI
Bangladesh	Diarrhoea	4.7	[4.2-5.3]		Malawi	Diarrhoea	21.7	[20.7-22.6]
Bangladesh	Fever	n/a	n/a		Malawi	Fever	28.9	[27.7-30.0]
Bangladesh	ARI	3.0	[2.6-3.5]		Malawi	ARI	5.4	[4.9-5.9]
Bangladesh	Any IMCI	7.4	[6.8-8.1]		Malawi	Any IMCI	41.6	[40.4-42.9]
DR Congo	Diarrhoea	14.0	[13.0-15.1]		Nepal	Diarrhoea	10.4	[9.2-11.8]
DR Congo	Fever	28.1	[26.5-29.8]		Nepal	Fever	n/a	n/a
DR Congo	ARI	3.6	[3.0-4.4]		Nepal	ARI	1.5	[1.1-1.9]
DR Congo	Any IMCI	35.7	[34.0-37.4]		Nepal	Any IMCI	11.5	[10.2-13.0]
Haiti	Diarrhoea	21.2	[19.7-22.8]		Senegal	Diarrhoea	16.4	[15.6-17.2]
Haiti	Fever	n/a	n/a		Senegal	Fever	18.7	[17.9-19.7]
Haiti	ARI	10.0	[8.9-11.3]		Senegal	ARI	4.6	[4.2-5.1]
Haiti	Any IMCI	28.4	[26.7-30.1]		Senegal	Any IMCI	28.9	[27.7-30.0]
Kenya	Diarrhoea	14.3	[13.5-15.1]		Tanzania	Diarrhoea	11.8	[10.9-12.8]
Kenya	Fever	17.1	[16.2-18.1]		Tanzania	Fever	17.9	[16.7-19.2]
Kenya	ARI	1.7	[1.5-2.0]		Tanzania	ARI	3.7	[3.2-4.3]
Kenya	Any IMCI	26.4	[25.3-27.5]		Tanzania	Any IMCI	26.0	[24.7-27.4]

** Any IMCI: Diarrhoea, Fever and/or ARI/Pneumonia*
ARI: Acute respiratory infections
n/a: Non-applicable (non-available)

Table S3. Sources of care of child illness

Country	Source of care	%	95% CI	Country	Source of care	%	95% CI
Bangladesh	Public hospital	4.3	[2.9-6.2]	Malawi	Public hospital	8.6	[7.3- 10.2]
Bangladesh	Public health center	13.7	[11.2-16.8]	Malawi	Public health center	44.9	[42.8-47.1]
Bangladesh	Private hospital	1.9	[1.0-3.5]	Malawi	Private hospital	4.9	[4.1-5.9]
Bangladesh	Private health center	24.1	[20.6-28.1]	Malawi	Private health center	3.4	[2.7-4.2]
Bangladesh	Pharmacy/depot	43.1	[38.9-47.4]	Malawi	Pharmacy/depot	6.2	[5.5-7.1]
Bangladesh	CHW/spec. voluntary	1.2	[0.6-2.6]	Malawi	CHW/spec. voluntary	4.3	[3.7-5.2]
Bangladesh	NGO	0.8	[0.3-2.5]	Malawi	NGO	0.1	[0.1-0.2]
Bangladesh	Non-specified	50.7	[46.1-55.2]	Malawi	Non-specified	65.4	[63.7-67.1]
DR Congo	Public hospital	2.8	[2.0- 4.0]	Nepal	Public hospital	1.8	[1.0-3.4]
DR Congo	Public health center	17.5	[14.7-20.6]	Nepal	Public health center	12.6	[9.9-16.0]
DR Congo	Private hospital	5.0	[4.0-6.2]	Nepal	Private hospital	2.8	[1.5-5.4]
DR Congo	Private health center	4.1	[3.3- 5.2]	Nepal	Private health center	23.8	[19.7-28.4]
DR Congo	Pharmacy/depot	16.4	[14.2- 18.9]	Nepal	Pharmacy/depot	19.1	[15.2-23.9]
DR Congo	CHW/spec. voluntary	3.0	[2.4-3.9]	Nepal	CHW/spec. voluntary	0.1	[0.4-2.2]
DR Congo	NGO	-	-	Nepal	NGO	0.0	-
DR Congo	Non-specified	10.5	[9.0-12.2]	Nepal	Non-specified	41.6	[37.1-46.2]
Haiti	Public hospital	7.0	[5.3-9.3]	Senegal	Public hospital	2.0	[1.5-2.6]
Haiti	Public health center	12.9	[11.0-15.1]	Senegal	Public health center	38.4	[36.7-40.2]
Haiti	Private hospital	1.9	[1.1-3.0]	Senegal	Private hospital	1.9	[1.4-2.5]
Haiti	Private health center	6.5	[5.0-8.4]	Senegal	Private health center	1.2	[0.8-1.6]
Haiti	Pharmacy/depot	3.7	[2.7-5.1]	Senegal	Pharmacy/depot	6.9	[5.9-8.0]
Haiti	CHW/spec. voluntary	1.7	[1.1-2.5]	Senegal	CHW/spec. voluntary	0.7	[0.4-1.0]
Haiti	NGO	1.6	[1.1-2.4]	Senegal	NGO	-	-
Haiti	Non-specified	38.6	[35.4-42.0]	Senegal	Non-specified	46.2	[44.4-47.9]
Kenya	Public hospital	13.4	[11.8- 15.1]	Tanzania	Public hospital	3.9	[2.7-5.8]
Kenya	Public health center	26.9	[25.2-28.6]	Tanzania	Public health center	29.7	[25.9-33.8]
Kenya	Private hospital	8.7	[7.4-10.1]	Tanzania	Private hospital	0.6	[0.2-1.5]
Kenya	Private health center	6.2	[5.3-7.3]	Tanzania	Private health center	5.3	[3.7-7.5]
Kenya	Pharmacy/depot	14.0	[12.5-15.6]	Tanzania	Pharmacy/depot	27.7	[24.4-31.2]
Kenya	CHW/spec. voluntary	0.2	[0.1-0.4]	Tanzania	CHW/spec. voluntary	4.2	[2.9-5.9]
Kenya	NGO	0.2	[0.1-0.6]	Tanzania	NGO	0	-
Kenya	Non-specified	56.0	[53.9-58.1]	Tanzania	Non-specified	3.4	[2.2-5.2]

NGO: Non-Governmental Organization
CHW/spec. voluntary: Community Health Worker/Specialized voluntary/Fieldworker

Table S4. Health facility service readiness for child care by component and category of facility

Country	Facility category	%	95% CI	Country	Facility category	%	95% CI
**OVERALL FACILITY READINESS FOR CHILD ILLNESS MANAGEMENT-SCORE**							
Bangladesh	Public hospital	72.5	[69.7-75.3]	Malawi	Public hospital	76.2	[73.9-78.6]
	Public health center	53.4	[52.6-54.2]	Malawi	Public health center	65.8	[64.4-67.1]
	Private hospital	58.2	[55.0-61.4]	Malawi	Private hospital	69.8	[65.2-74.4]
	Private health center	60.2	[56.6-63.8]	Malawi	Private health center	54.4	[52.8-55.9]
	Total	55.0	[54.2-55.8]	Malawi	Total	61.5	[60.5-62.6]
DR Congo	Public hospital	65.4	[64.0-66.9]	Nepal	Public hospital	69.4	[67.5-71.2]
	Public health center	61.9	[60.8-62.9]	Nepal	Public health center	58.3	[57.6-59.0]
	Private hospital	66.7	[65.3-68.1]	Nepal	Private hospital	56.2	[54.5-57.8]
	Private health center	63.4	[61.6-65.1]	Nepal	Private health center	24.0	[20.6-27.4]
	Total	64.0	[63.3-64.7]	Nepal	Total	58.1	[57.4-58.7]
Haiti	Public hospital	66.6	[61.3-71.9]	Senegal	Public hospital	70.8	[65.0-76.5]
	Public health center	61.2	[59.5-62.9]	Senegal	Public health center	64.1	[62.3-65.9]
	Private hospital	62.6	[58.9-66.3]	Senegal	Private hospital	51.0	[40.1-61.9]
	Private health center	54.8	[53.4-56.1]	Senegal	Private health center	43.4	[37.8-49.0]
	Total	57.9	[56.8-58.9]	Senegal	Total	61.0	[59.2-62.8]
Kenya	Public hospital	74.5	[73.5-75.5]	Tanzania	Public hospital	70.6	[68.3-72.8]
	Public health center	68.6	[68.0-69.2]	Tanzania	Public health center	61.5	[60.5-62.5]
	Private hospital	77.0	[74.0-80.0]	Tanzania	Private hospital	72.1	[70.0-74.2]
	Private health center	59.1	[57.9-60.3]	Tanzania	Private health center	55.7	[53.3-58.1]
	Total	65.7	[65.1-66.3]	Tanzania	Total	62.3	[61.4-63.2]
**AVAILABILITY OF CHILD HEALTH SERVICE - SCORE**							
Bangladesh	Public hospital	89.6	[86.5-92.6]	Malawi	Public hospital	93.8	[90.9-96.6]
	Public health center	66.4	[65.3-67.4]	Malawi	Public health center	89.6	[87.8-91.3]
	Private hospital	69.0	[64.5-73.5]	Malawi	Private hospital	83.3	[77.4-89.2]
	Private health center	63.9	[59.0-68.8]	Malawi	Private health center	66.6	[64.1-69.1]
	Total	67.4	[66.3-68.4]	Malawi	Total	79.2	[77.6-80.8]
DR Congo	Public hospital	77.3	[75.4-79.3]	Nepal	Public hospital	93.8	[91.7-95.8]
	Public health center	82.0	[80.6-83.4]	Nepal	Public health center	75.0	[73.9-76.0]
	Private hospital	76.8	[74.9-78.6]	Nepal	Private hospital	73.0	[70.6-75.5]
	Private health center	81.0	[78.8-83.3]	Nepal	Private health center	13.2	[7.6-18.7]
	Total	79.6	[78.7-80.5]	Nepal	Total	74.8	[73.7-75.8]
Haiti	Public hospital	83.0	[75.9-90.1]	Senegal	Public hospital	83.6	[73.6-93.5]
	Public health center	82.6	[80.5-84.7]	Senegal	Public health center	90.5	[88.8-92.3]
	Private hospital	71.8	[66.8-76.7]	Senegal	Private hospital	71.9	[59.4-84.4]
	Private health center	69.1	[67.2-70.9]	Senegal	Private health center	58.9	[51.5-66.3]
	Total	73.9	[72.5-75.3]	Senegal	Total	85.1	[82.9-87.2]

Country	Facility category	%	95% CI	Country	Facility category	%	95% CI
Kenya	Public hospital	97.1	[95.8-98.4]	Tanzania	Public hospital	86.8	[83.9-89.7]
	Public health center	91.9	[91.1-92.7]	Tanzania	Public health center	82.0	[80.8-83.2]
	Private hospital	90.8	[86.9-94.7]	Tanzania	Private hospital	88.4	[85.6-91.3]
	Private health center	70.2	[68.2-72.3]	Tanzania	Private health center	67.3	[64.0-70.6]
	Total	83.7	[82.7-84.7]	Tanzania	Total	79.8	[78.7-81.0]
**AVAILABILITY OF SUPPLIES FOR SICK CHILDREN - SCORE**							
Bangladesh	Public hospital	77.3	[72.6-82.0]	Malawi	Public hospital	74.8	[70.6-79.0]
	Public health center	52.3	[51.2-53.4]	Malawi	Public health center	66.7	[65.0-68.4]
	Private hospital	57.5	[54.1-61.0]	Malawi	Private hospital	70.6	[65.6-75.6]
	Private health center	63.8	[59.2-68.4]	Malawi	Private health center	57.0	[55.1-59.0]
	Total	54.5	[53.4-55.5]	Malawi	Total	63.1	[61.8-64.3]
DR Congo	Public hospital	58.4	[56.5-60.3]	Nepal	Public hospital	71.5	[69.0-73.9]
	Public health center	58.5	[57.2-59.8]	Nepal	Public health center	63.8	[62.9-64.6]
	Private hospital	57.3	[55.4-59.1]	Nepal	Private hospital	57.2	[55.3-59.1]
	Private health center	59.1	[57.0-61.1]	Nepal	Private health center	34.4	[29.7-39.1]
	Total	58.3	[57.5-59.2]	Nepal	Total	62.6	[61.8-63.4]
Haiti	Public hospital	62.4	[57.0-67.8]	Senegal	Public hospital	67.9	[60.4-75.3]
	Public health center	59.9	[57.6-62.1]	Senegal	Public health center	64.9	[62.9-66.9]
	Private hospital	56.5	[51.5-61.6]	Senegal	Private hospital	55.8	[47.0-64.7]
	Private health center	51.9	[50.3-53.6]	Senegal	Private health center	44.7	[39.2-50.1]
	Total	55.1	[53.9-56.4]	Senegal	Total	61.8	[59.9-63.7]
Kenya	Public hospital	64.7	[62.7-66.8]	Tanzania	Public hospital	68.2	[65.2-71.2]
	Public health center	58.8	[57.8-59.8]	Tanzania	Public health center	62.6	[61.3-64.0]
	Private hospital	67.2	[62.9-71.4]	Tanzania	Private hospital	70.9	[68.0-73.8]
	Private health center	48.6	[47.4-49.7]	Tanzania	Private health center	52.2	[49.6-54.8]
	Total	55.6	[54.9-56.3]	Tanzania	Total	61.7	[60.6-62.8]
**AVAILABILITY OF DIAGNOSTICS FOR SICK CHILDREN - SCORE**							
Bangladesh	Public hospital	n/a	[n/a-n/a]	Malawi	Public hospital	86.0	[79.7-92.3]
	Public health center	n/a	[n/a-n/a]	Malawi	Public health center	39.8	[37.7-41.9]
	Private hospital	n/a	[n/a-n/a]	Malawi	Private hospital	81.8	[74.6-89.0]
	Private health center	n/a	[n/a-n/a]	Malawi	Private health center	43.4	[40.7-46.2]
	Total	n/a	[n/a-n/a]	Malawi	Total	46.6	[44.8-48.5]
DR Congo	Public hospital	92.2	[90.2-94.3]	Nepal	Public hospital	n/a	n/a
	Public health center	69.4	[66.3-72.6]	Nepal	Public health center	n/a	n/a
	Private hospital	94.7	[93.0-96.4]	Nepal	Private hospital	n/a	n/a
	Private health center	77.0	[72.5-81.5]	Nepal	Private health center	n/a	n/a
	Total	81.6	[80.0-83.3]	Nepal	Total	n/a	n/a
Haiti	Public hospital	90.0	[82.6-97.4]	Senegal	Public hospital	97.4	[92.2-100.0]

Country	Facility category	%	95% CI	Country	Facility category	%	95% CI
	Public health center	37.6	[32.4-42.8]	Senegal	Public health center	50.9	[48.9-52.9]
	Private hospital	89.5	[83.1-95.9]	Senegal	Private hospital	66.7	[48.2-85.1]
	Private health center	57.7	[54.0-61.5]	Senegal	Private health center	46.8	[39.0-54.6]
	Total	56.0	[53.1-58.9]	Senegal	Total	52.8	[50.6-55.0]
Kenya	Public hospital	16.3	[13.6-18.9]	Tanzania	Public hospital	96.3	[93.5-99.2]
	Public health center	23.1	[21.8-24.5]	Tanzania	Public health center	46.3	[42.8-49.7]
	Private hospital	33.5	[28.1-38.9]	Tanzania	Private hospital	97.7	[95.7-99.8]
	Private health center	25.0	[23.5-26.4]	Tanzania	Private health center	73.8	[69.0-78.7]
	Total	23.4	[22.5-24.4]	Tanzania	Total	63.6	[61.1-66.1]
**AVAILABILITY OF MEDICINES FOR SICK CHILDREN - SCORE**							
Bangladesh	Public hospital	56.0	[49.9-62.0]	Malawi	Public hospital	72.3	[69.1-75.5]
	Public health center	46.5	[45.1-47.8]	Malawi	Public health center	63.4	[61.4-65.4]
	Private hospital	55.0	[48.2-61.8]	Malawi	Private hospital	67.7	[62.4-73.0]
	Private health center	55.6	[50.4-60.8]	Malawi	Private health center	59.7	[57.7-61.8]
	Total	48.1	[46.8-49.4]	Malawi	Total	62.5	[61.2-63.9]
DR Congo	Public hospital	66.8	[64.4-69.1]	Nepal	Public hospital	69.7	[66.8-72.6]
	Public health center	54.4	[52.3-56.4]	Nepal	Public health center	55.3	[54.4-56.3]
	Private hospital	71.5	[69.0-74.0]	Nepal	Private hospital	50.0	[46.2-53.8]
	Private health center	59.5	[56.0-63.0]	Nepal	Private health center	8.3	[4.2-12.4]
	Total	61.9	[60.6-63.2]	Nepal	Total	54.5	[53.5-55.6]
Haiti	Public hospital	62.5	[52.9-72.1]	Senegal	Public hospital	67.5	[58.0-77.1]
	Public health center	56.9	[54.0-59.7]	Senegal	Public health center	52.6	[49.1-56.0]
	Private hospital	69.4	[63.7-75.2]	Senegal	Private hospital	33.3	[12.0-54.7]
	Private health center	50.7	[48.6-52.8]	Senegal	Private health center	33.1	[24.7-41.4]
	Total	54.6	[52.9-56.2]	Senegal	Total	49.8	[46.7-53.0]
Kenya	Public hospital	70.9	[69.3-72.5]	Tanzania	Public hospital	69.4	[66.1-72.6]
	Public health center	61.8	[60.9-62.7]	Tanzania	Public health center	53.5	[52.1-54.8]
	Private hospital	82.6	[77.9-87.3]	Tanzania	Private hospital	73.3	[70.9-75.7]
	Private health center	62.5	[60.9-64.2]	Tanzania	Private health center	57.6	[54.5-60.7]
	Total	63.7	[62.9-64.6]	Tanzania	Total	58.3	[57.1-59.5]
**TRAINING & SUPERVISION OF HEALTH PERSONNEL IN CHILD ILLNESS MANAGEMENT-SCORE**							
Bangladesh	Public hospital	56.9	[49.5-64.2]	Malawi	Public hospital	61.0	[54.2-67.8]
	Public health center	46.8	[45.3-48.3]	Malawi	Public health center	47.8	[45.2-50.5]
	Private hospital	43.3	[38.9-47.8]	Malawi	Private hospital	53.0	[46.4-59.6]
	Private health center	54.6	[49.4-59.7]	Malawi	Private health center	34.6	[32.1-37.1]
	Total	47.5	[46.1-48.9]	Malawi	Total	43.0	[41.2-44.8]
DR Congo	Public hospital	54.2	[51.2-57.2]	Nepal	Public hospital	42.9	[37.5-48.2]
	Public health center	50.0	[47.8-52.3]	Nepal	Public health center	37.6	[35.5-39.6]

Country	Facility category	%	95% CI		Country	Facility category	%	95% CI
	Private hospital	57.9	[54.9-60.9]		Nepal	Private hospital	57.6	[52.3-62.8]
	Private health center	50.3	[46.7-53.9]		Nepal	Private health center	68.0	[54.8-81.3]
	Total	52.8	[51.4-54.2]		Nepal	Total	42.1	[40.3-44.0]
Haiti	Public hospital	52.8	[45.1-60.4]		Senegal	Public hospital	52.0	[36.2-67.7]
	Public health center	48.9	[46.1-51.7]		Senegal	Public health center	49.2	[45.8-52.5]
	Private hospital	50.2	[44.5-55.8]		Senegal	Private hospital	30.2	[12.7-47.7]
	Private health center	43.6	[41.4-45.7]		Senegal	Private health center	32.1	[24.3-39.8]
	Total	46.1	[44.5-47.7]		Senegal	Total	46.3	[43.2-49.3]
Kenya	Public hospital	n/a	n/a		Tanzania	Public hospital	51.6	[46.2-57.0]
	Public health center	n/a	n/a		Tanzania	Public health center	50.4	[47.9-52.8]
	Private hospital	n/a	n/a		Tanzania	Private hospital	49.6	[44.5-54.8]
	Private health center	n/a	n/a		Tanzania	Private health center	42.2	[38.3-46.0]
	Total	n/a	n/a		Tanzania	Total	48.5	[46.7-50.3]

Table S5. Health facility process quality by component and category of facility

Country	Facility category	%	95%CI	Country	Facility category	%	95%CI
OVERALL PROCESS QUALITY DURING CLINICAL ENCOUNTER-SCORE							
Country	Facility category	%	95%CI	Country	Facility category	%	95%CI
DR Congo	Public hospital	60.8	[59.7-61.8]	Nepal	Public hospital	66.4	[65.2-67.6]
	Public health center	53.0	[51.9-54.1]	Nepal	Public health center	50.5	[49.4-51.7]
	Private hospital	58.2	[56.9-59.5]	Nepal	Private hospital	64.6	[63.1-66.1]
	Private health center	55.4	[53.8-56.9]	Nepal	Private health center	-	-
	Total	56.8	[56.2-57.4]	Nepal	Total	58.5	[57.7-59.3]
Haiti	Public hospital	59.2	[57.0-61.5]	Senegal	Public hospital	58.4	[55.5-61.3]
	Public health center	48.6	[47.1-50.1]	Senegal	Public health center	52.1	[51.1-53.2]
	Private hospital	60.7	[58.7-62.7]	Senegal	Private hospital	50.6	[44.1-57.1]
	Private health center	55.0	[54.1-55.9]	Senegal	Private health center	42.2	[39.7-44.7]
	Total	54.8	[54.0-55.5]	Senegal	Total	51.6	[50.7-52.6]
Malawi	Public hospital	59.6	[58.1-61.0]	Tanzania	Public hospital	68.7	[67.6-69.8]
	Public health center	53.3	[52.6-53.9]	Tanzania	Public health center	56.5	[55.8-57.2]
	Private hospital	63.6	[61.3-66.0]	Tanzania	Private hospital	67.4	[66.2-68.7]
	Private health center	54.2	[53.2-55.2]	Tanzania	Private health center	55.5	[54.1-57.0]
	Total	54.9	[54.4-55.4]	Tanzania	Total	60.1	[59.6-60.6]
ASSESSMENT OF CHILD HEALTH DURING CLINICAL ENCOUNTER - SCORE							
DR Congo	Public hospital	64.6	[63.3-66.0]	Nepal	Public hospital	70.5	[69.2-71.8]
	Public health center	57.6	[56.3-58.9]	Nepal	Public health center	54.5	[53.2-55.8]
	Private hospital	61.2	[59.6-62.7]	Nepal	Private hospital	70.3	[68.6-72.0]
	Private health center	57.8	[56.0-59.6]	Nepal	Private health center	-	-
	Total	60.5	[59.7-61.2]	Nepal	Total	62.9	[62.0-63.8]
Haiti	Public hospital	62.7	[60.1-65.2]	Senegal	Public hospital	50.8	[46.9-54.7]
	Public health center	54.1	[52.3-56.0]	Senegal	Public health center	48.3	[46.9-49.7]
	Private hospital	63.8	[61.7-65.9]	Senegal	Private hospital	46.5	[35.9-57.1]
	Private health center	61.4	[60.3-62.5]	Senegal	Private health center	37.8	[34.5-41.0]
	Total	60.2	[59.3-61.0]	Senegal	Total	47.4	[46.2-48.6]
Malawi	Public hospital	62.8	[60.9-64.8]	Tanzania	Public hospital	79.5	[78.3-80.7]
	Public health center	58.0	[57.2-58.8]	Tanzania	Public health center	70.6	[69.8-71.3]
	Private hospital	68.7	[66.4-71.0]	Tanzania	Private hospital	79.4	[78.0-80.8]
	Private health center	59.1	[57.9-60.3]	Tanzania	Private health center	68.6	[66.8-70.4]
	Total	59.6	[58.9-60.2]	Tanzania	Total	73.2	[72.6-73.7]
PHYSICAL EXAM OF CHILD HEALTH DURING CLINICAL ENCOUNTER - SCORE							
Country	Facility category	%	95%CI	Country	Facility category	%	95%CI
DR Congo	Public hospital	66.9	[65.7-68.2]	Nepal	Public hospital	675.2	[63.9-66.6]
	Public health center	55.5	[54.2-56.8]	Nepal	Public health center	49.0	[47.7-50.2]

Country	Facility category	%	95%CI	Country	Facility category	%	95%CI
	Private hospital	64.9	[63.4-66.5]	Nepal	Private hospital	63.6	[61.7-65.4]
	Private health center	58.7	[56.7-60.6]	Nepal	Private health center	-	-
	Total	61.4	[60.7-62.2]	Nepal	Total	57.2	[56.3-58.1]
Haiti	Public hospital	61.3	[58.7-64.0]	Senegal	Public hospital	85.2	[82.3-88.0]
	Public health center	51.7	[50.1-53.4]	Senegal	Public health center	72.8	[71.4-74.2]
	Private hospital	65.8	[63.2-68.4]	Senegal	Private hospital	70.1	[63.8-76.5]
	Private health center	55.1	[54.0-56.2]	Senegal	Private health center	63.8	[58.6-69.0]
	Total	56.4	[55.6-57.2]	Senegal	Total	72.9	[71.6-74.2]
Malawi	Public hospital	65.9	[63.8-68.0]	Tanzania	Public hospital	58.7	[57.4-60.1]
	Public health center	50.7	[49.8-51.6]	Tanzania	Public health center	43.2	[42.4-44.0]
	Private hospital	67.1	[64.3-69.8]	Tanzania	Private hospital	58.9	[57.4-60.5]
	Private health center	53.5	[52.4-54.6]	Tanzania	Private health center	44.5	[42.8-46.2]
	Total	54.2	[53.5-54.9]	Tanzania	Total	48.3	[47.7-49.0]
**COUNSELING OF CHILD CARETAKER DURING CLINICAL ENCOUNTER - SCORE**							
**Country**	**Facility category**	**%**	**95%CI**	**Country**	**Facility category**	**%**	**95%CI**
DR Congo	Public hospital	27.4	[25.2-29.6]	Nepal	Public hospital	62.5	[59.9-65.0]
	Public health center	30.2	[28.0-32.4]	Nepal	Public health center	39.8	[37.9-41.8]
	Private hospital	28.9	[26.2-31.6]	Nepal	Private hospital	61.6	[58.6-64.7]
	Private health center	31.1	[27.7-34.5]	Nepal	Private health center	-	-
	Total	29.2	[28.0-30.5]	Nepal	Total	51.6	[50.2-53.1]
Haiti	Public hospital	45.9	[41.0-50.7]	Senegal	Public hospital	34.6	[26.2-43.0]
	Public health center	25.2	[22.6-27.8]	Senegal	Public health center	17.8	[15.8-19.8]
	Private hospital	44.8	[41.2-48.4]	Senegal	Private hospital	24.2	[6.4-42.1]
	Private health center	35.2	[33.4-37.0]	Senegal	Private health center	9.3	[5.0-13.5]
	Total	35.4	[34.0-36.7]	Senegal	Total	18.6	[16.7-20.4]
Malawi	Public hospital	26.5	[23.3-29.7]	Tanzania	Public hospital	54.1	[51.6-56.6]
	Public health center	30.0	[28.5-31.4]	Tanzania	Public health center	39.8	[38.5-41.1]
	Private hospital	40.1	[35.3-44.8]	Tanzania	Private hospital	48.5	[45.7-51.3]
	Private health center	31.7	[29.6-33.8]	Tanzania	Private health center	36.2	[33.3-39.0]
	Total	30.8	[29.7-31.9]	Tanzania	Total	43.2	[42.1-44.2]
**TREATMENT PROVIDED/PRESCRIBED TO CHILD DURING CLINICAL ENCOUNTER - SCORE**							
**Country**	**Facility category**	**%**	**95%CI**	**Country**	**Facility category**	**%**	**95%CI**
DR Congo	Public hospital	71.1	[68.5-73.6]	Nepal	Public hospital	49.3	[47.0-51.7]
	Public health center	58.9	[56.3-61.6]	Nepal	Public health center	67.8	[66.2-69.4]
	Private hospital	63.3	[59.9-66.7]	Nepal	Private hospital	49.9	[47.5-52.3]
	Private health center	69.4	[66.0-72.9]	Nepal	Private health center	-	-
	Total	65.1	[63.6-66.5]	Nepal	Total	58.2	[56.9-59.4]
Haiti	Public hospital	58.7	[56.3-61.2]	Senegal	Public hospital	29.5	[20.9-38.2]

Country	Facility category	%	95%CI	Country	Facility category	%	95%CI
	Public health center	54.3	[52.4-56.3]	Senegal	Public health center	40.6	[37.8-43.5]
	Private hospital	35.1	[32.5-37.7]	Senegal	Private hospital	22.7	[4.8-40.7]
	Private health center	74.6	[73.5-75.6]	Senegal	Private health center	20.0	[12.9-27.1]
	Total	63.2	[62.2-64.2]	Senegal	Total	37.1	[34.6-39.7]
Malawi	Public hospital	78.5	[75.4-81.6]	Tanzania	Public hospital	75.6	[73.5-77.6]
	Public health center	81.7	[80.3-83.0]	Tanzania	Public health center	70.8	[69.5-72.0]
	Private hospital	68.8	[64.9-72.7]	Tanzania	Private hospital	72.9	[70.6-75.2]
	Private health center	74.9	[72.8-77.0]	Tanzania	Private health center	69.7	[67.0-72.3]
	Total	78.6	[77.5-79.6]	Tanzania	Total	71.8	[70.9-72.7]

Table S6. Effective coverage estimates of IMCI by place of residence

Country	Cascade items	Urban				Rural			
		%	lb 95%CI	ub 95%CI	N	%	lb 95%CI	ub 95%CI	N
Bangladesh	Sick child	100.0			224	100.0			423
Bangladesh	Careseeking (any)	88.6	83.1	92.4		88.1	84.1	91.2	
Bangladesh	Service contact	84.9	78.9	89.3		77.3	72.5	81.5	
Bangladesh	Readiness-adjusted contact	26.3	21.0	31.6		22.9	19.7	26.1	
Bangladesh	Crude coverage	88.2	82.6	92.2		81.0	76.5	84.9	
Bangladesh	Quality-adjusted coverage	n/a	n/a	n/a		n/a	n/a	n/a	
DR Congo	Sick child	100.0			2048	100.0			5213
DR Congo	Careseeking (any)	57.8	53.3	62.2		51.8	48.3	55.2	
DR Congo	Service contact	39.3	34.7	44.1		35.2	31.2	39.3	
DR Congo	Readiness-adjusted contact	20.6	18.2	23.1		17.8	14.8	20.9	
DR Congo	Crude coverage	32.4	29.0	36.0		22.3	19.1	25.9	
DR Congo	Quality-adjusted coverage	12.9	10.9	14.9		8.6	6.7	10.4	
Haiti	Sick child	100.0			515	100.0			1151
Haiti	Careseeking (any)	51.6	45.5	57.6		35.5	31.9	39.4	
Haiti	Service contact	44.5	38.8	50.3		29.6	26.1	33.3	
Haiti	Readiness-adjusted contact	21.7	18.1	25.3		14.7	12.8	16.5	
Haiti	Crude coverage	48.5	43.2	54.0		34.8	31.1	38.7	
Haiti	Quality-adjusted coverage	16.4	13.3	19.4		10.7	9.1	12.3	
Kenya	Sick child	100.0			1668	100.0			3180
Kenya	Careseeking (any)	69.7	65.7	73.5		68.0	65.9	70.2	
Kenya	Service contact	69.0	64.9	72.7		66.2	64.0	68.4	
Kenya	Readiness-adjusted contact	36.4	33.4	39.5		38.4	36.9	40.0	
Kenya	Crude coverage	46.5	42.6	50.5		45.8	43.6	48.0	
Kenya	Quality-adjusted coverage	n/a	n/a	n/a		n/a	n/a	n/a	
Malawi	Sick child	100.0			936	100.0			5755
Malawi	Careseeking (any)	64.6	59.6	69.2		71.2	69.5	72.9	
Malawi	Service contact	62.3	57.0	67.3		64.7	62.9	66.4	
Malawi	Readiness-adjusted contact	42.8	39.2	46.3		40.6	39.4	41.9	
Malawi	Crude coverage	63.0	57.9	67.7		63.0	61.1	64.9	
Malawi	Quality-adjusted coverage	30.5	27.6	33.4		28.3	27.2	29.4	
Nepal	Sick child	100.0			307	100.0			280
Nepal	Careseeking (any)	58.3	51.8	64.5		62.2	54.6	69.2	
Nepal	Service contact	55.8	48.6	62.7		61.6	54.0	68.6	
Nepal	Readiness-adjusted contact	14.8	12.2	17.3		16.8	14.1	19.4	
Nepal	Crude coverage	43.2	36.3	50.4		42.9	36.2	49.8	
Nepal	Quality-adjusted coverage	6.7	4.5	8.9		9.2	6.6	11.7	

Country	Cascade items	Urban				Rural			
		%	lb 95%CI	ub 95%CI	N	%	lb 95%CI	ub 95%CI	N
Senegal	Sick child	100.0			2310	100.0			4751
Senegal	Careseeking (any)	61.8	58.3	65.2		46.8	44.9	48.8	
Senegal	Service contact	51.7	48.5	54.8		42.3	40.3	44.2	
Senegal	Readiness-adjusted contact	31.0	28.9	33.1		25.5	24.2	26.8	
Senegal	Crude coverage	30.6	27.8	33.5		28.0	26.2	29.8	
Senegal	Quality-adjusted coverage	13.9	12.4	15.4		12.5	11.6	13.3	
Tanzania	Sick child	100.0			613	100.0			1834
Tanzania	Careseeking (any)	80.5	76.6	83.9		77.9	75.1	80.5	
Tanzania	Service contact	70.9	66.5	74.9		53.5	50.0	56.9	
Tanzania	Readiness-adjusted contact	37.4	34.3	40.4		24.7	22.3	27.2	
Tanzania	Crude coverage	59.4	54.8	63.8		38.3	35.2	41.6	
Tanzania	Quality-adjusted coverage	26.5	23.9	29.0		15.6	13.7	17.4	
n/a: Non available									

Table S7. Effective coverage estimates of IMCI by wealth quintile

Country	Cascade items	Richest				Poorest			
		%	lb 95%CI	ub 95%CI	N	%	lb 95%CI	ub 95%CI	N
Bangladesh	Sick child	100.0			226	100.0			421
Bangladesh	Careseeking (any)	88.4	82.9	92.3		88.1	83.9	91.3	
Bangladesh	Service contact	83.7	77.9	88.2		77.1	72.0	81.5	
Bangladesh	Readiness-adjusted contact	30.7	25.9	35.4		20.4	17.3	23.6	
Bangladesh	Crude coverage	84.9	79.1	89.3		81.9	77.1	85.8	
Bangladesh	Quality-adjusted coverage	n/a	n/a	n/a		n/a	n/a	n/a	
DR Congo	Sick child	100.0			1489	100.0			5772
DR Congo	Careseeking (any)	60.5	56.1	64.7		50.8	47.5	54.2	
DR Congo	Service contact	42.1	37.2	47.1		34.0	30.3	37.9	
DR Congo	Readiness-adjusted contact	22.1	19.6	24.7		17.3	14.5	20.1	
DR Congo	Crude coverage	33.7	30.0	37.6		22.4	19.4	25.7	
DR Congo	Quality-adjusted coverage	13.6	11.5	15.8		8.5	6.8	10.2	
Haiti	Sick child	100.0			462	100.0			1204
Haiti	Careseeking (any)	49.6	43.2	56.1		37.3	33.4	41.2	
Haiti	Service contact	42.1	36.5	47.9		31.5	27.8	35.5	
Haiti	Readiness-adjusted contact	20.9	17.6	24.1		15.4	13.2	17.6	
Haiti	Crude coverage	45.1	39.5	50.8		37.2	33.3	41.2	
Haiti	Quality-adjusted coverage	16.1	13.3	18.9		11.1	9.3	12.9	
Kenya	Sick child	100.0			1540	100.0			3308
Kenya	Careseeking (any)	70.5	66.7	74.0		67.4	65.2	70.0	
Kenya	Service contact	69.7	65.9	73.2		65.6	63.4	67.7	
Kenya	Readiness-adjusted contact	37.1	34.2	39.9		38.1	36.5	39.6	
Kenya	Crude coverage	44.3	40.6	48.0		47.3	45.1	49.6	
Kenya	Quality-adjusted coverage	n/a	n/a	n/a		n/a	n/a	n/a	
Malawi	Sick child	100.0			2254	100.0			4437
Malawi	Careseeking (any)	70.1	67.5	72.6		70.5	68.6	72.3	
Malawi	Service contact	64.5	61.8	67.1		64.3	62.3	66.3	
Malawi	Readiness-adjusted contact	42.4	40.5	44.3		40.2	38.7	41.7	
Malawi	Crude coverage	65.5	62.7	68.2		61.8	59.7	63.9	
Malawi	Quality-adjusted coverage	30.2	28.6	31.8		27.8	26.6	29.0	
Nepal	Sick child	100.0			147	100.0			440
Nepal	Careseeking (any)	60.6	50.9	69.5		59.0	52.9	64.8	
Nepal	Service contact	58.3	47.6	68.3		57.3	51.1	63.3	
Nepal	Readiness-adjusted contact	12.5	8.8	16.1		16.8	14.5	19.1	
Nepal	Crude coverage	39.3	30.0	49.4		44.9	39.1	50.9	
Nepal	Quality-adjusted coverage	4.3	1.4	7.2		9.0	6.9	11.1	

Country	Cascade items	Richest				Poorest			
		%	lb 95%CI	ub 95%CI	N	%	lb 95%CI	ub 95%CI	N
Senegal	Sick child	100.0			1587	100.0			5474
Senegal	Careseeking (any)	63.5	59.9	67.0		47.0	45.0	48.9	
Senegal	Service contact	54.1	50.7	57.4		41.7	39.9	43.5	
Senegal	Readiness-adjusted contact	32.7	30.4	35.0		25.0	23.8	26.2	
Senegal	Crude coverage	30.8	27.9	33.9		28.0	26.4	29.8	
Senegal	Quality-adjusted coverage	13.9	12.3	15.5		12.6	11.8	13.4	
Tanzania	Sick child	100.0			960	100.0			1487
Tanzania	Careseeking (any)	81.5	78.0	84.5		77.0	74.0	79.7	
Tanzania	Service contact	67.6	63.4	71.4		53.0	49.3	56.7	
Tanzania	Readiness-adjusted contact	34.8	31.7	38.0		24.5	22.0	27.0	
Tanzania	Crude coverage	54.4	49.6	59.0		38.4	35.2	41.7	
Tanzania	Quality-adjusted coverage	23.6	21.1	26.2		15.7	13.8	17.7	

Table S8. Effective coverage estimates of IMCI by education level of the mother/caretaker

Country	Cascade items	Secondary				Primary				None			
		%	lb 95%CI	ub 95%CI	N	%	lb 95%CI	ub 95%CI	N	%	lb 95%CI	ub 95%CI	N
Bangladesh	Sick child	100.0			403	100.0			197	100			47
Bangladesh	Careseeking (any)	88.9	85.0	91.9		88.0	81.5	92.5		82.8	67.6	91.8	
Bangladesh	Service contact	80.8	76.1	84.8		77.1	69.8	83.0		75.3	59.7	86.2	
Bangladesh	Readiness-adjusted contact	27.8	24.2	31.4		16.5	12.2	20.7		19.9	10.6	29.3	
Bangladesh	Crude coverage	82.6	78.0	86.4		85.1	78.9	89.7		76.4	60.6	87.2	
Bangladesh	Quality-adjusted coverage	n/a	n/a	n/a		n/a	n/a	n/a		n/a	n/a	n/a	
DR Congo	Sick child	100.0			2686	100.0			2998	100.0			1562
DR Congo	Careseeking (any)	57.1	53.4	60.8		52.9	48.6	57.2		49.3	44.4	54.3	
DR Congo	Service contact	39.6	36.0	43.3		35.5	31.2	40.1		32.4	27.0	38.4	
DR Congo	Readiness-adjusted contact	21.2	18.9	23.5		17.5	14.5	20.6		16.1	12.3	19.9	
DR Congo	Crude coverage	30.3	27.2	33.6		23.2	20.0	26.7		22.6	17.4	28.9	
DR Congo	Quality-adjusted coverage	12.0	10.3	13.8		9.0	7.2	10.7		8.5	5.4	11.6	
Haiti	Sick child	100.0			645	100.0			696	100.0			325
Haiti	Careseeking (any)	48.1	43.1	53.1		37.9	33.3	42.6		34.4	28.6	40.6	
Haiti	Service contact	42.4	37.6	47.4		31.1	26.9	35.6		27.4	21.8	33.9	
Haiti	Readiness-adjusted contact	21.4	18.4	24.4		15.0	12.4	17.6		12.8	9.6	16.0	
Haiti	Crude coverage	45.3	40.8	49.9		35.6	30.8	40.7		36.9	30.7	43.7	
Haiti	Quality-adjusted coverage	16.7	14.1	19.3		10.0	8.0	11.9		10.0	6.9	13.1	
Kenya	Sick child	100.0			2023	100.0			1792	100.0			1033
Kenya	Careseeking (any)	71.1	68.0	74.0		67.1	64.1	70.3		61.9	56.9	66.6	
Kenya	Service contact	70.0	66.8	72.9		65.2	62.2	68.1		60.9	55.9	65.7	
Kenya	Readiness-adjusted contact	38.6	36.2	41.0		36.4	34.3	38.5		38.1	34.6	41.7	
Kenya	Crude coverage	43.9	41.0	46.8		48.5	45.5	51.6		48.0	43.3	52.7	
Kenya	Quality-adjusted coverage	n/a	n/a	n/a		n/a	n/a	n/a		n/a	n/a	n/a	
Malawi	Sick child	100.0			1339	100.0			4583	100.0			769
Malawi	Careseeking (any)	69.7	66.2	73.1		71.5	69.7	73.3		65.0	60.6	69.2	
Malawi	Service contact	64.8	61.0	68.4		65.5	63.6	67.3		57.5	53.2	61.7	
Malawi	Readiness-adjusted contact	42.4	39.7	45.0		41.0	39.6	42.4		37.9	35.0	40.8	
Malawi	Crude coverage	65.6	61.5	69.5		63.2	61.2	65.2		57.8	53.3	62.0	
Malawi	Quality-adjusted coverage	30.3	27.9	32.8		28.6	27.4	29.7		25.8	23.4	28.3	
Nepal	Sick child	100.0			236	100.0			220	100.0			131
Nepal	Careseeking (any)	60.0	52.6	67.0		57.3	49.6	64.6		62.3	52.2	71.4	
Nepal	Service contact	59.4	52.1	66.3		53.7	44.2	62.9		61.0	50.6	70.4	
Nepal	Readiness-adjusted contact	15.3	12.2	18.4		15.6	12.0	19.2		15.2	10.9	19.5	
Nepal	Crude coverage	39.9	33.0	47.1		46.1	37.6	53.0		43.9	33.9	54.5	
Nepal	Quality-adjusted coverage	7.4	4.7	10.0		7.6	4.7	10.4		7.6	3.7	11.4	

Country	Cascade items	Secondary				Primary				None			
		%	lb 95%CI	ub 95%CI	N	%	lb 95%CI	ub 95%CI	N	%	lb 95%CI	ub 95%CI	N
Senegal	Sick child	100.0			1163	100.0			1461	100.0			4436
Senegal	Careseeking (any)	62.7	57.9	67.2		59.4	56.0	62.7		46.9	44.5	49.2	
Senegal	Service contact	54.9	50.3	59.4		51.4	48.0	54.8		41.0	38.9	43.0	
Senegal	Readiness-adjusted contact	32.9	30.1	35.7		31.0	28.6	33.3		24.7	23.3	26.0	
Senegal	Crude coverage	32.7	28.9	36.9		32.8	29.1	36.6		26.4	24.7	28.2	
Senegal	Quality-adjusted coverage	14.9	12.9	16.9		15.0	13.1	16.9		11.7	10.9	12.6	
Tanzania	Sick child	100.0			485	100.0			1451	100.0			511
Tanzania	Careseeking (any)	83.1	78.4	87.0		78.6	75.5	81.4		75.5	70.1	80.2	
Tanzania	Service contact	70.4	64.6	75.5		59.7	56.2	63.1		45.1	40.0	51.4	
Tanzania	Readiness-adjusted contact	36.6	31.9	40.7		28.8	26.3	31.4		20.9	17.0	31.8	
Tanzania	Crude coverage	55.7	49.8	61.6		45.5	42.2	48.7		32.0	26.7	37.8	
Tanzania	Quality-adjusted coverage	24.9	21.3	28.5		19.1	17.2	21.0		12.6	9.6	15.5	

Table S9. Effective coverage estimates of IMCI by age of the mother/caretaker

Country	Cascade items	Younger age group (15-29 years)				Older age group (30-49 years)			
		%	lb 95%CI	ub 95%CI	N	%	lb 95%CI	ub 95%CI	N
Bangladesh	Sick child	100.0			521	100.0			126
Bangladesh	Careseeking (any)	89.7	86.2	92.4		81.7	73.0	88.1	
Bangladesh	Service contact	80.9	76.5	84.6		72.4	62.8	80.2	
Bangladesh	Readiness-adjusted contact	24.5	21.4	27.7		20.6	14.7	26.5	
Bangladesh	Crude coverage	84.3	80.3	87.6		76.9	66.7	84.7	
Bangladesh	Quality-adjusted coverage	n/a	n/a	n/a		n/a	n/a	n/a	
DR Congo	Sick child	100.0			3541	100.0			3706
DR Congo	Careseeking (any)	54.6	51.3	57.9		53.7	50.4	57.0	
DR Congo	Service contact	36.3	33.1	39.8		37.3	33.6	41.2	
DR Congo	Readiness-adjusted contact	19.3	16.8	21.8		18.6	16.1	21.1	
DR Congo	Crude coverage	27.3	24.0	30.8		25.4	22.5	28.5	
DR Congo	Quality-adjusted coverage	10.7	8.9	12.5		9.9	8.3	11.5	
Haiti	Sick child	100.0			889	100.0			777
Haiti	Careseeking (any)	39.8	35.4	44.3		43.5	39.4	47.7	
Haiti	Service contact	34.6	30.6	38.9		35.8	31.8	40.0	
Haiti	Readiness-adjusted contact	16.8	14.4	19.1		17.9	15.4	20.3	
Haiti	Crude coverage	37.5	33.7	41.4		42.7	38.2	47.3	
Haiti	Quality-adjusted coverage	11.9	10.0	13.7		13.9	11.5	16.2	
Kenya	Sick child	100.0			2844	100.0			2004
Kenya	Careseeking (any)	68.5	65.9	70.9		69.1	66.0	72.0	
Kenya	Service contact	66.9	64.3	69.3		68.0	64.9	70.8	
Kenya	Readiness-adjusted contact	38.4	36.4	40.4		36.5	34.3	38.7	
Kenya	Crude coverage	47.4	44.7	50.0		43.9	40.9	47.0	
Kenya	Quality-adjusted coverage	n/a	n/a	n/a		n/a	n/a	n/a	
Malawi	Sick child	100.0			4307	100.0			2384
Malawi	Careseeking (any)	71.6	69.8	73.3		68.2	65.5	70.9	
Malawi	Service contact	66.4	64.5	68.3		60.8	58.0	63.5	
Malawi	Readiness-adjusted contact	41.9	40.5	43.3		39.1	37.2	41.1	
Malawi	Crude coverage	64.6	62.6	66.6		60.1	57.3	62.9	
Malawi	Quality-adjusted coverage	29.2	28.1	30.3		27.4	25.7	29.0	
Nepal	Sick child	100.0			451	100.0			136
Nepal	Careseeking (any)	59.5	53.8	65.1		59.4	49.3	68.7	
Nepal	Service contact	57.5	51.1	63.6		58.1	47.9	67.6	
Nepal	Readiness-adjusted contact	14.7	12.6	16.9		17.5	12.9	22.1	
Nepal	Crude coverage	41.8	35.7	48.2		47.3	37.5	57.3	
Nepal	Quality-adjusted coverage	9.7	5.5	14.0		6.8	5.0	8.6	

Country	Cascade items	Younger age group (15-29 years)				Older age group (30-49 years)			
		%	lb 95%CI	ub 95%CI	N	%	lb 95%CI	ub 95%CI	N
Senegal	Sick child	100.0			3670	100.0			3391
Senegal	Careseeking (any)	53.1	50.6	55.7		51.9	49.5	54.2	
Senegal	Service contact	46.2	43.9	48.4		45.5	43.3	47.7	
Senegal	Readiness-adjusted contact	27.8	26.3	29.4		27.3	25.8	28.8	
Senegal	Crude coverage	29.6	27.5	31.8		28.3	26.3	30.5	
Senegal	Quality-adjusted coverage	13.5	12.4	14.7		12.5	11.5	13.5	
Tanzania	Sick child	100.0			1413	100.0			1034
Tanzania	Careseeking (any)	78.7	75.7	81.5		78.6	75.2	81.7	
Tanzania	Service contact	60.2	56.6	63.7		56.1	51.7	60.4	
Tanzania	Readiness-adjusted contact	29.5	27.1	32.0		26.7	23.9	29.6	
Tanzania	Crude coverage	45.7	42.5	49.0		42.6	38.4	46.9	
Tanzania	Quality-adjusted coverage	19.5	17.7	21.4		17.6	15.2	19.9	

Table S10. Effective coverage estimates of IMCI by age of the child

Country	Cascade items	Younger age group (0-23 months)				Older age group (24-59 months)			
		%	lb 95%CI	ub 95%CI	N	%	lb 95%CI	ub 95%CI	N
Bangladesh	Sick child	100.0			388	100.0			259
Bangladesh	Careseeking (any)	88.9	84.7	92.0		87.3	82.2	91.1	
Bangladesh	Service contact	80.4	75.5	84.6		77.6	71.7	82.5	
Bangladesh	Readiness-adjusted contact	26.5	23.1	30.0		19.8	15.7	24.0	
Bangladesh	Crude coverage	80.4	75.5	84.6		86.4	80.8	90.5	
Bangladesh	Quality-adjusted coverage	n/a	n/a	n/a		n/a	n/a	n/a	
DR Congo	Sick child	100.0			3216	100.0			4045
DR Congo	Careseeking (any)	57.0	53.3	60.7		51.8	48.7	55.0	
DR Congo	Service contact	40.3	36.6	44.2		33.9	30.6	37.5	
DR Congo	Readiness-adjusted contact	20.6	18.4	22.8		17.7	15.1	20.2	
DR Congo	Crude coverage	29.1	26.0	32.5		24.0	21.1	27.2	
DR Congo	Quality-adjusted coverage	11.4	9.6	13.2		9.3	7.8	10.9	
Haiti	Sick child	100.0			872	100.0			794
Haiti	Careseeking (any)	42.0	38.2	45.9		41.0	36.2	46.0	
Haiti	Service contact	36.2	32.5	40.0		34.1	29.6	38.9	
Haiti	Readiness-adjusted contact	18.1	15.9	20.2		16.5	13.7	19.2	
Haiti	Crude coverage	39.7	35.9	43.5		40.2	36.0	44.4	
Haiti	Quality-adjusted coverage	13.1	11.3	14.9		12.5	10.2	14.8	
Kenya	Sick child	100.0			2488	100.0			2360
Kenya	Careseeking (any)	67.0	64.3	69.5		70.5	67.7	73.1	
Kenya	Service contact	65.7	63.1	68.3		68.8	66.1	71.5	
Kenya	Readiness-adjusted contact	38.2	36.2	40.2		37.1	35.0	39.1	
Kenya	Crude coverage	47.0	44.2	49.9		45.1	42.3	47.9	
Kenya	Quality-adjusted coverage	n/a	n/a	n/a		n/a	n/a	n/a	
Malawi	Sick child	100.0			3224	100.0			3467
Malawi	Careseeking (any)	70.3	68.3	72.1		70.5	68.2	72.7	
Malawi	Service contact	65.8	63.8	67.8		63.0	60.6	65.4	
Malawi	Readiness-adjusted contact	41.5	40.0	42.9		40.4	38.7	42.1	
Malawi	Crude coverage	63.5	61.2	65.7		62.6	60.1	64.9	
Malawi	Quality-adjusted coverage	28.6	27.4	29.8		28.5	27.1	30.0	
Nepal	Sick child	100.0			293	100.0			294
Nepal	Careseeking (any)	58.2	50.9	65.2		60.8	53.4	67.7	
Nepal	Service contact	56.0	48.6	63.2		59.2	51.2	66.8	
Nepal	Readiness-adjusted contact	14.3	11.9	16.7		16.5	13.3	19.7	
Nepal	Crude coverage	32.5	26.2	39.4		53.7	46.0	61.2	
Nepal	Quality-adjusted coverage	6.3	4.2	8.4		8.7	6.0	11.3	

Country	Cascade items	Younger age group (0-23 months)				Older age group (24-59 months)			
		%	lb 95%CI	ub 95%CI	N	%	lb 95%CI	ub 95%CI	N
Senegal	Sick child	100.0			3741	100.0			3320
Senegal	Careseeking (any)	55.3	53.1	57.4		49.4	46.8	52.0	
Senegal	Service contact	48.5	46.2	50.8		42.8	40.4	45.3	
Senegal	Readiness-adjusted contact	29.2	27.7	30.6		25.7	24.1	27.4	
Senegal	Crude coverage	29.8	27.8	31.9		28.0	25.9	30.1	
Senegal	Quality-adjusted coverage	13.7	12.6	14.7		12.3	11.3	13.3	
Tanzania	Sick child	100.0			1308	100.0			1139
Tanzania	Careseeking (any)	79.5	76.3	82.3		77.8	74.7	80.7	
Tanzania	Service contact	63.5	59.9	66.9		53.0	48.9	57.0	
Tanzania	Readiness-adjusted contact	29.8	27.3	32.4		26.8	24.2	29.4	
Tanzania	Crude coverage	49.0	45.5	52.7		39.3	35.5	43.4	
Tanzania	Quality-adjusted coverage	19.8	17.7	21.8		17.6	15.5	19.7	

Table S11. Effective coverage estimates of IMCI by sex of the child

Country	Cascade items	Male				Female			
		%	lb 95%CI	ub 95%CI	N	%	lb 95%CI	ub 95%CI	N
Bangladesh	Sick child	100.0			376	100.0			271
Bangladesh	Careseeking (any)	90.4	86.5	93.2		85.2	79.6	89.5	
Bangladesh	Service contact	80.5	75.4	84.7		77.5	71.7	82.5	
Bangladesh	Readiness-adjusted contact	25.3	21.6	28.9		21.7	17.7	25.8	
Bangladesh	Crude coverage	84.0	79.3	87.7		81.4	75.6	86.0	
Bangladesh	Quality-adjusted coverage	n/a	n/a	n/a		n/a	n/a	n/a	
DR Congo	Sick child	100.0			3646	100.0			3615
DR Congo	Careseeking (any)	53.5	50.3	56.7		54.8	51.4	58.1	
DR Congo	Service contact	35.9	32.4	39.5		37.6	34.0	41.4	
DR Congo	Readiness-adjusted contact	18.6	15.8	21.4		19.3	17.2	21.4	
DR Congo	Crude coverage	26.4	22.8	30.3		26.2	23.6	29.1	
DR Congo	Quality-adjusted coverage	10.0	8.0	11.9		10.6	9.1	12.0	
Haiti	Sick child	100.0			850	100.0			816
Haiti	Careseeking (any)	39.8	35.6	44.2		43.2	38.5	48.1	
Haiti	Service contact	34.0	30.2	38.1		36.3	31.7	41.1	
Haiti	Readiness-adjusted contact	39.1	34.9	43.5		40.7	36.0	45.7	
Haiti	Crude coverage	16.0	13.8	18.2		18.6	15.7	21.5	
Haiti	Quality-adjusted coverage	11.3	9.5	13.2		14.3	11.9	16.7	
Kenya	Sick child	100.0			2484	100.0			2364
Kenya	Careseeking (any)	68.9	66.2	71.5		68.5	65.6	71.1	
Kenya	Service contact	67.6	64.8	70.2		66.9	64.1	69.6	
Kenya	Readiness-adjusted contact	38.3	36.3	40.3		36.9	34.9	39.0	
Kenya	Crude coverage	48.1	45.4	50.8		43.9	41.1	46.8	
Kenya	Quality-adjusted coverage	n/a	n/a	n/a		n/a	n/a	n/a	
Malawi	Sick child	100.0			3405	100.0			3286
Malawi	Careseeking (any)	71.1	69.2	73.0		69.6	67.4	71.7	
Malawi	Service contact	65.1	63.1	67.0		63.6	61.3	65.9	
Malawi	Readiness-adjusted contact	41.6	40.2	43.0		40.2	38.6	41.8	
Malawi	Crude coverage	63.4	62.3	66.4		61.6	59.3	63.8	
Malawi	Quality-adjusted coverage	29.4	28.2	30.6		27.7	26.4	28.9	
Nepal	Sick child	100.0			321	100.0			266
Nepal	Careseeking (any)	61.3	54.4	67.8		57.4	49.8	64.7	
Nepal	Service contact	60.0	53.1	66.5		54.9	47.1	62.5	
Nepal	Readiness-adjusted contact	15.6	12.9	18.4		15.1	12.3	17.9	
Nepal	Crude coverage	46.0	39.0	53.2		39.8	33.0	47.0	
Nepal	Quality-adjusted coverage	6.8	4.7	9.0		8.3	5.5	11.0	

Country	Cascade items	Male				Female			
		%	lb 95%CI	ub 95%CI	N	%	lb 95%CI	ub 95%CI	N
Senegal	Sick child	100.0			3647	100.0			3414
Senegal	Careseeking (any)	53.5	51.3	55.7		51.4	48.9	53.9	
Senegal	Service contact	46.9	44.7	49.0		44.7	42.5	46.9	
Senegal	Readiness-adjusted contact	28.4	26.9	29.8		26.7	25.2	28.2	
Senegal	Crude coverage	29.9	27.8	32.0		28.0	26.1	30.0	
Senegal	Quality-adjusted coverage	13.5	12.5	14.6		12.5	11.5	13.4	
Tanzania	Sick child	100.0			1226	100.0			1221
Tanzania	Careseeking (any)	78.6	75.6	81.3		78.8	75.8	81.5	
Tanzania	Service contact	59.0	55.3	62.6		58.0	54.4	61.7	
Tanzania	Readiness-adjusted contact	29.3	26.9	31.7		27.5	24.7	30.2	
Tanzania	Crude coverage	45.0	41.4	48.7		43.8	40.2	47.5	
Tanzania	Quality-adjusted coverage	19.4	17.4	21.4		18.0	16.0	20.1	

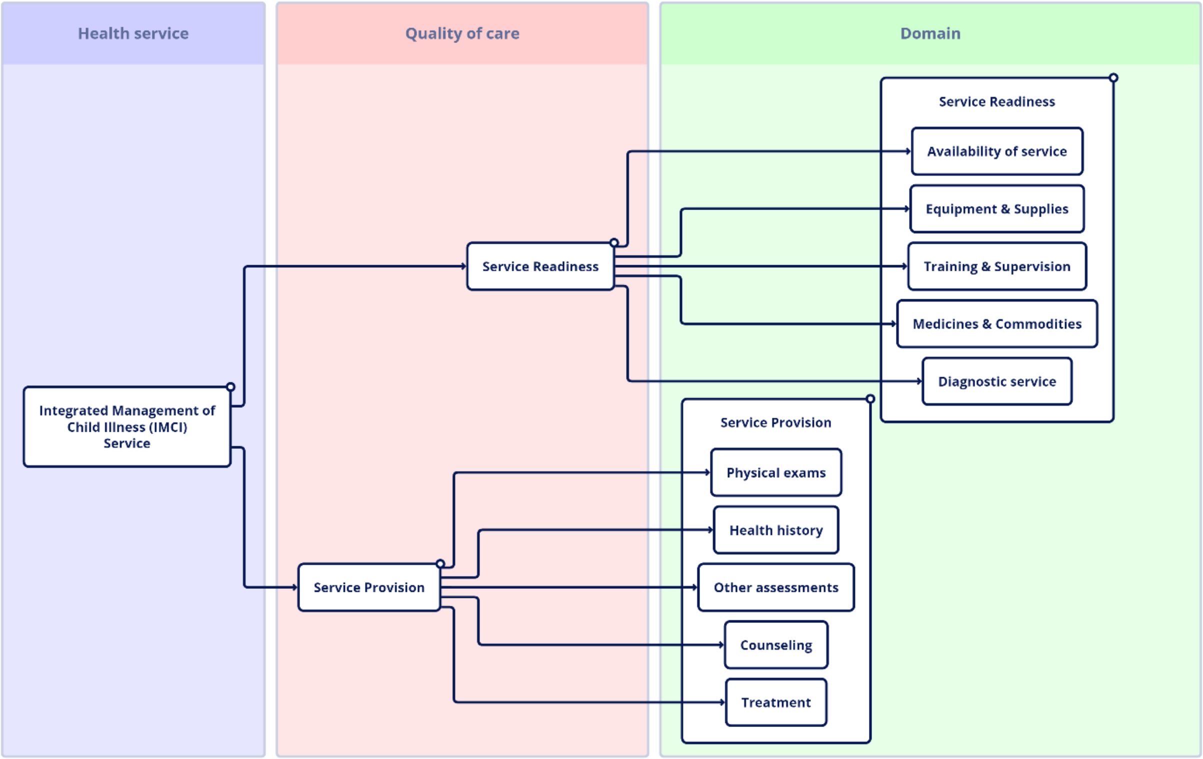

Figure S1. Structure and components readiness and process quality
